# Liquid Crystal Elastomer‐Based Haptic Pixel Arrays at Your Fingertips for Advanced Human–Machine Interfaces

**DOI:** 10.1002/adma.74546

**Published:** 2026-08-09

**Authors:** Tom Bruining, Enjun Lin, Yu Zou, Meike Heldoorn, Pengrong Lyu, Samuël A. M. Weima, Astrid M. L. Kappers, Myrthe A. Plaisier, Satoshi Aya, Danqing Liu

**Affiliations:** ^1^ Human Interactive Materials (HIM), Department of Chemical Engineering and Chemistry Eindhoven University of Technology Eindhoven The Netherlands; ^2^ South China Advanced Institute for Soft Matter Science and Technology (AISMST), School of Emergent Soft Matter South China University of Technology Guangzhou China; ^3^ Guangdong Provincial Key Laboratory of Functional and Intelligent Hybrid Materials and Devices Guangdong Basic Research Center of Excellence for Energy and Information Polymer Materials South China University of Technology Guangzhou China; ^4^ Human‐Technology Interaction (HTI), Department of Industrial Engineering & Innovation Sciences Eindhoven University of Technology Eindhoven The Netherlands; ^5^ Robotics (RBT), Department of Mechanical Engineering Eindhoven University of Technology Eindhoven The Netherlands; ^6^ School of Materials and Energy Guangdong University of Technology Guangzhou China

**Keywords:** dynamic topographies, human–machine interfaces, liquid crystal polymer coatings, perceptual performance, surface haptics, theoretical modeling

## Abstract

The sense of touch is underutilized in our digital lives, especially when compared to the prevalence and complexity of information transfer through our visual and auditory interactions with devices; the impact of this is profound as touch plays a major role in our perception of our world, and its omission in our commonly used devices limits our accessibility to the digital space, particularly for blind and visually impaired users. Fortunately, the stimuli‐responsive shape‐morphing properties of liquid crystal elastomers (LCEs) promise a solution: in our work, we mechanically program an LCE bilayer coating to achieve durable, high‐amplitude switchable surface protrusions, or tactile pixels, which we assemble into a digital platform for generating tactile images and haptic feedback. We further characterize and describe our tactile pixel design by microscopy techniques and simulations that use the neo‐classical theory of rubber elasticity. We demonstrate safe and effective electronically addressable activation of individual LCE pixels within seconds, accompanied by a psychophysical evaluation of tactile line orientation discrimination in which the performance of our LCE tactile pixels is comparable to swell paper, a standard tactile graphic medium. Thereby, we establish LCEs as a contender for the next‐generation dynamic tactile displays.

## Graphical abstract

We mechanically program liquid crystal elastomer coatings as a stimuli‐responsive and digitally addressable platform for refreshable tactile displays; we validate the perceptual performance of our dynamic device matches conventional static tactile media. We envision LCE active coatings will enable the next‐generation dynamic tactile interfaces for an accessible and equal virtual world.

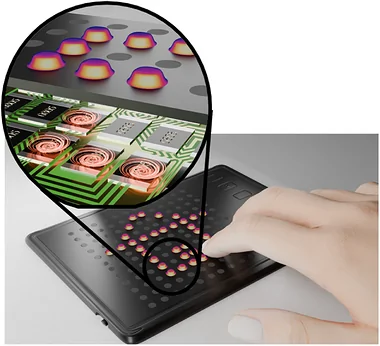

## 1 Introduction

Digital information has become a defining feature of communication in modern society, shaping how we learn, work, innovate, and socialize [1]. However, despite being an integral part of our activities as human beings, the overwhelming majority of digital media is only accessible through two of our five senses, namely visually or auditory, for instance, smartphones, computer monitors, and television [2]. Consequently, our level of immersion in digital activities is limited, since many of our senses are not engaged in the virtual space [3, 4]. For people with blindness and visual impairments, interaction with digital technology is even more restricted [2, 5]. The development of new haptic technologies that allow devices to interact with our sense of touch has great potential to increase user experience and improve the inclusivity of many digital devices, facilitating technology adoption for a wider audience.

Tactile images, for example those used in books for people with visual impairments, offer a powerful nonvisual alternative to static visual images; by encoding shapes as raised lines and regions as textured relief patterns, they provide tangible representations of objects, scenes, or spatial information [6]. This allows for more effective transfer of visual information for people with visual impairments compared to verbal descriptions in the form of braille or audio. Traditionally, such graphics are produced on static substrates including embossed paper, thermoformed plastics, or swell paper. Although effective, these media are inherently non‐refreshable, restricting users to fixed content or the need to reprint new information; this is unsuitable for integration with digital devices. To address this limitation, refreshable tactile displays based on mechanically‐actuated pin arrays have been developed; in these systems, grids of vertically moving pins, typically driven by piezoelectric actuators, form dynamic relief patterns, and are also able to display Braille when needed [7]. However, pin displays are mechanically complex, bulky, and costly, and their reliance on numerous moving components makes them susceptible to wear [8, 9, 10]. Consequently, widespread access to refreshable tactile graphics remains restricted, causing disparities in digital accessibility.

In our work, we address digital inaccessibility and inequality by introducing a coating‐based refreshable tactile display platform that can be digitally programmed to reversibly generate protrusions, acting as haptic pixels, which outline selectable tactile images; we present the concept for our tactile display device in Figure 1a. The combined features of this device are made possible by our use of liquid crystal elastomers (LCEs), a class of stimuli‐responsive soft materials widely explored in the scientific community as actuators due to its shape‐morphing behavior; this originates from collective molecular motion driven by LCE material phase changes [11, 12, 13, 14]. Liquid crystal (LC) phases typically involve molecules being aligned in a given axis, known as the director (*n*), with the degree to which molecules are aligned with the *n* being known as the order parameter (*S*); an *S* of 1 denotes molecules being perfectly‐aligned with the *n*, while an *S* of 0 denotes that the orientation of molecules is random. Loss of *S*, which can be triggered with heat, light, or electricity, induces contraction along, and expansion orthogonal to, the *n*, and vice versa [15, 16]. Therefore, unlike conventional electromechanical motor systems, LCE actuators can operate within a compact, single‐material monolithic architecture. Hence, LCE actuators are machine‐like materials that promise a solution for robust, scalable, and high‐resolution tactile interfaces [17, 18, 19, 20, 21].

**FIGURE 1:**
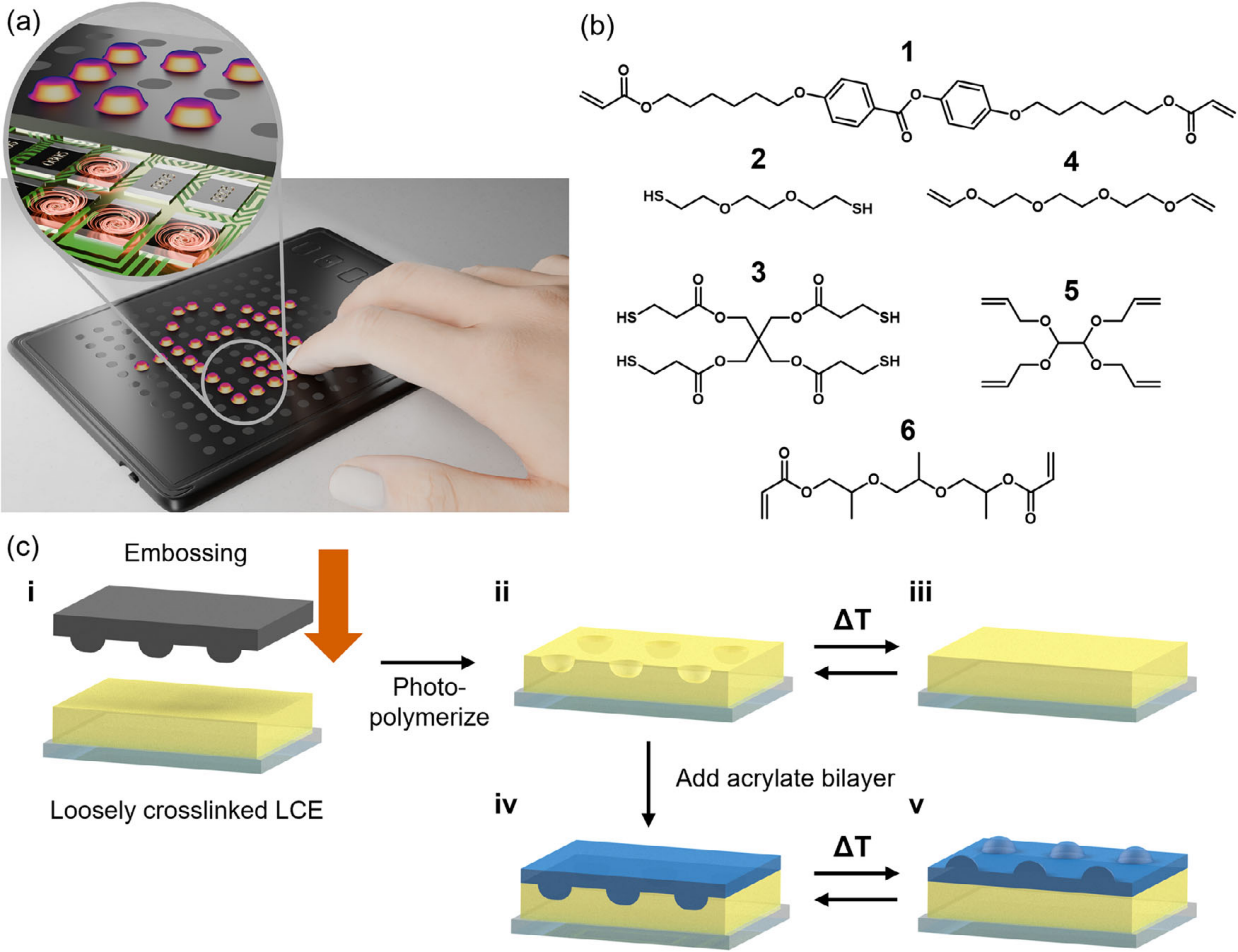
(a) Conceptual visualization of a tactile display screen featuring a responsive tactile LCE coating which is locally stimulated by heating resistors (heat is depicted in red in the zoomed‐in illustration) to display a tactile image. (b) Monomers used in the production of the LCE material (**1‐** **5**) and the acrylate layer (**2, 6**). (c) Schematic of the production process; (i) the loosely cross‐linked LCE is embossed with a stamp to create indents, (ii) the indentations are permanently programmed using a photopolymerization step, (iii) when heated, this material undergoes reversible actuation, returning to a flat state. (iv) An acrylate‐based bilayer is added to form the final topcoat, forming a flat surface at room temperature. (v) When the material is heated, the actuating LCE layer pushes the acrylate layer up to form protrusions.

Our approach builds on the well‐established two‐step cross‐linking strategy for mechanically programming LCEs [22, 23]. Utilizing this process, we fabricate LCE coatings with a passive acrylate bilayer, programmed to reversibly switch between a flat state and a state featuring high‐amplitude surface protrusions as a response to specified temperature changes in a safe range for human contact. Our integration of a bilayer in a novel way allows us to reach a near‐flat off‐state at room temperature, while creating a robust system that is able to produce sufficient actuation force for real‐world human interaction. We supplement our understanding of this order‐parameter‐change‐driven phenomenon by resolving the mesogen alignment and its contribution to overall LCE actuation. Based on our knowledge, we enable digital addressability of our LCE coatings by employing controlled Joule heating as a localized actuation strategy [24, 25, 26, 27]; by selectively increasing the temperature of defined regions, individual protrusions can be activated within seconds, enabling dynamic tactile pattern formation. We utilize this concept to build a proof‐of‐concept demonstrator using a resistor grid and validate the function of our LCE pixels by evaluating the human perceptual performance with our system in a psychophysical study of tactile line orientation discrimination, benchmarking the material against swell paper. Overall, our work positions LCEs as a promising material to enable next‐generation refreshable tactile displays for enhanced and equal access to the digital world.

## 2 Results and Discussion

### 2.1 Liquid Crystal Elastomers as a Tactile Screen

We produce our LCE‐based haptic pixel coatings through a two‐step cross‐linking strategy that enables mechanical programming of LC mesogen alignment and, consequently, controllable actuation [22, 23, 28]. In the first step, we react LC mesogen **1** with chain extender **2** and cross‐linker **3** (Figure 1b) through a base‐catalyzed Michael addition, yielding a loosely cross‐linked elastomeric network. While in this malleable, partially cured state, a 3D‐printed stamp is pressed into the film to introduce defined surface indentations and simultaneously impose alignment of the embedded LC mesogens along the applied deformation (Figure 2c(i)). Since we design the reaction mixture formulation with a molar excess of thiol groups to generate thiol‐terminated polymer chains after the initial cross‐linking stage, while including vinyls that do not react in the first stage, mechanically‐induced anisotropy can be permanently fixed via UV‐initiated thiol–ene radical chemistry; here, residual thiols react with vinyl monomers **4** and **5** (Figure 1b), completing the second cross‐linking step and establishing a two‐way shape‐memory response. Furthermore, incorporation of non‐mesogenic vinyl monomers in the reaction mixture dilutes the LC mesogen concentration, reducing the nematic–isotropic transition temperature (*T*_NI_) of the resulting LCE [29].

**FIGURE 2:**
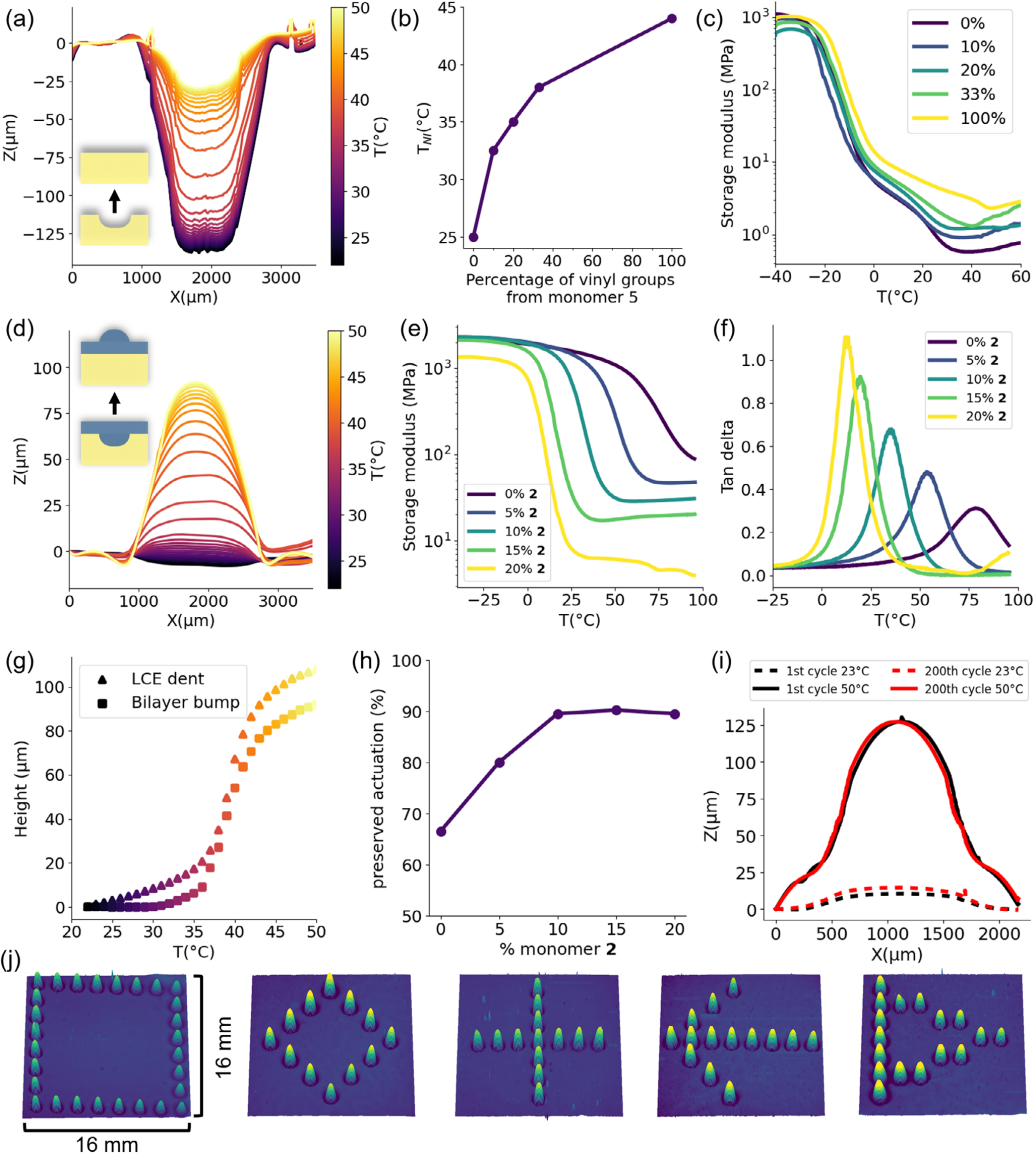
(a) 2D profile of the dent in the LCE layer at temperatures from 23°C to 50°C. (b) Nematic to isotropic transition temperature for mixtures with varying ratios of monomers **4** and **5**, expressed as the molar percentage of vinyl groups coming from monomer **5**. (c) Storage modulus over temperature for mixtures with varying ratios of monomers **4** and **5**. Expressed as the molar percentage of vinyl groups coming from monomer **5** in the legend. (d) 2D profile of the acrylate layer at temperatures from 23°C to 50°C. (e) Storage modulus and (f) tan delta of bilayer compositions consisting of monomer **6** with varying percentages of monomer **2**, which is indicated as **2** in the figure legend. (g) Vertical displacement of the deepest point of the indent in the LCE layer and the highest point of the protrusion in the acylate layer (with 10% monomer **2** composition) starting from room temperature and heating to 50°C. (h) Amount of actuation of the LCE bump preserved when actuating with bilayer compositions with varying percentages of monomer **2**. (i) 2D profile of tactile bump at room temperature and in an actuated state before and after 200 cycles of heating and cooling to demonstrate durability of the bilayer. (j) 3D images of actuated preprogrammed tactile icons.

Thereby, we initially program our LCEs to reversibly switch between an indented configuration at room temperature (Figure 1c(ii)) and a flattened state upon heating, driven by the thermally induced reduction of the nematic *S*. This actuation is characterized by interferometry. A two‐dimensional (2D) surface profile shows a 138 µm‐deep indentation at 22°C, that progressively decreases to 30 µm as the temperature is increased to 50°C (Figure 2a). To ensure human safety and practical usability for our thermally responsive LCE tactile interfaces, we adjusted the operational temperature window of our LCE material to between 37°C and 45°C. The upper temperature limit is dictated by skin tolerance: sustained contact at 45°C can cause discomfort and partial‐thickness burns within several hours [30]. The lower temperature bound is based on human finger surface temperature which, although variable across individuals and environments, typically remains below 37°C [31, 32]; if *T*_NI_ were below this value, incidental touch could trigger unintended actuation, rendering the unactuated state of the LCE active coating unstable.

**FIGURE 3:**
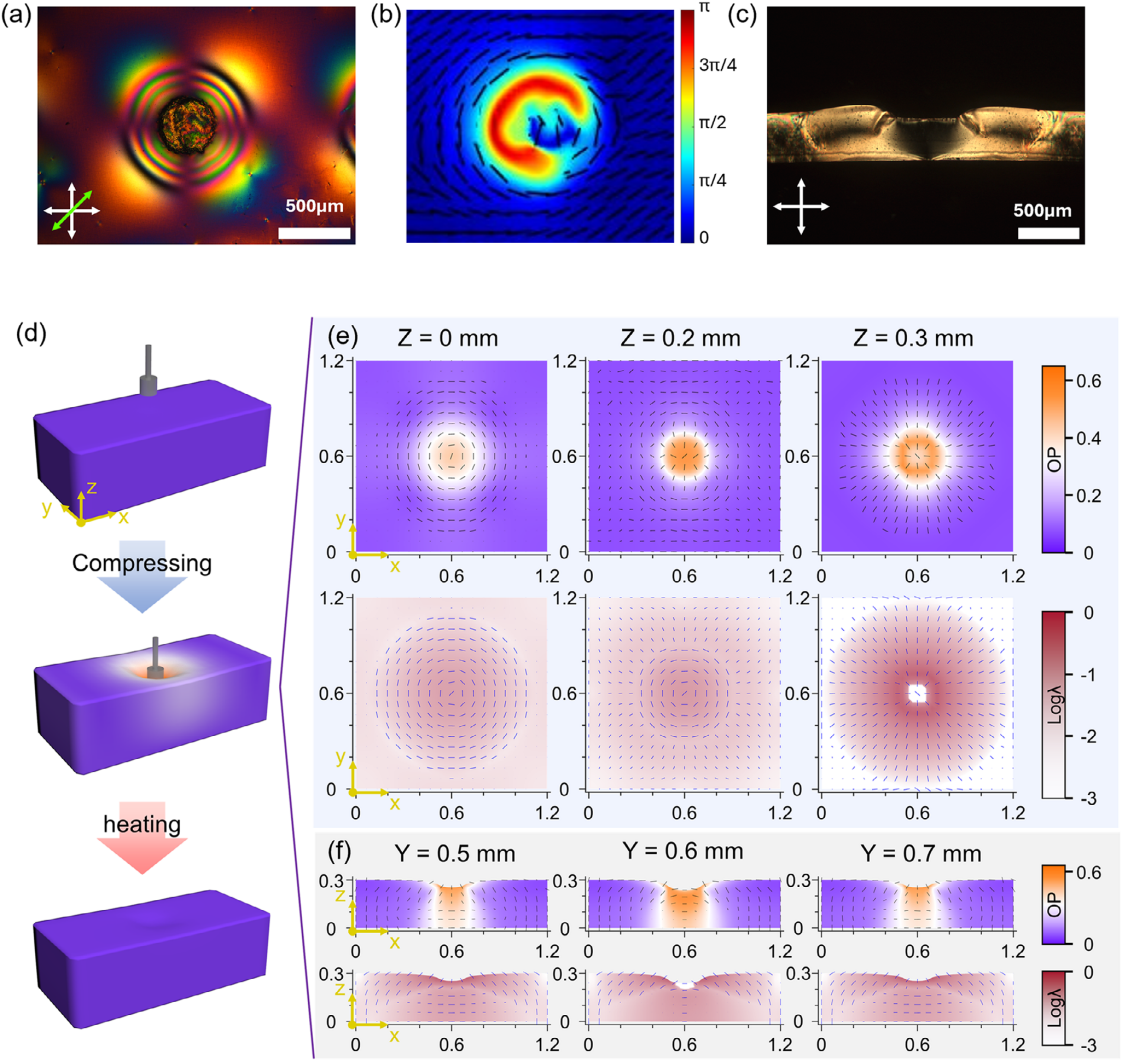
(a) Polarized optical microscopy (POM) image of an indentation in the LCE layer with a full wave plate inserted at a 45° angle between crossed polarizers. (b) Polscope image of an LCE indentation. (c) Cross section of an LCE indentation between crossed polarizers. (d) A cuboidal elastomer (1.2 mm × 1.2 mm × 0.3 mm) indented by a cylindrical probe. Compression generates a central surface depression and induces orientational order through stress‐order parameter (OP) coupling in Landau free energy term. Upon heating, the OP vanishes, the alignment becomes disordered, and the structure nearly recovers its initial shape. (e–f) Director fields as black arrows and OP distributions as the background color on the upper region together with the principal elongation directions (the eigenvector of the deformation gradient) as blue arrows and corresponding eigenvalues *λ* as the background color at the bottom region (e) in the xy cross sections at *z* = 0, 0.2, and 0.3 mm, and (f) in the xz cross sections at *y* = 0.5, 0.6, and 0.7 mm. All cross sections are taken with respect to the initial elastomer geometry (undeformed grid). Coordinates (*x*, *y*, *z*) are expressed in millimetres.

By varying the relative concentrations of vinyl monomers **4** and **5**, while maintaining a constant molar fraction of vinyl functional groups, we systematically adjusted *T*_NI_: exclusive use of monomer **5** yielded a *T*_NI_ of 44°C, whereas monomer **4** alone reduced *T*_NI_ to 25°C. Interpolating between these limits enabled identification of a formulation where monomer **5** provides a third of the vinyl groups with a *T*_NI_ of approximately 40°C (Figure 2b and Figure S1), exhibiting minimal actuation below 37°C and expressing the majority of its deformation prior to reaching 45°C. Besides affecting the actuation temperature, an increase in modulus is observed when increasing the ratio of monomer **5**, confirming the formation of networks with a higher cross‐link density (Figure 2c, full dynamic mechanical analysis (DMA) results including loss modulus and tan delta are provided in Figure S2).

To translate the deformation of the LCE layer into tactile protrusions, a flexible material based on diacrylate monomer **6** and dithiol monomer **2** was filled into the cavities of the programmed LCE surface and photopolymerized (Figure 1b,c(iv)). The resulting LCE bilayer laminate remains fully flat at room temperature, with less than 5 µm variation along the *z*‐axis (Figure 2d). Upon heating to 50°C, the induced deformation of the LCE layer generates an upward deflection of the passive, yet compliant, top‐coating, transforming the pre‐programmed indentations into raised surface features (Figures 1c(iv,v) and 2d). To find the optimal composition of the acrylate layer, the weight percentage of dithiol **2** with respect to diacrylate monomer **6** was varied. Dithiols are known to act as chain transfer agents in free radical polymerizations, where increased concentrations decrease the modulus and glass transition temperature (*T*_g_) in the final material (Figure 2e,f) [33]. Directly comparing the amount of actuation in µm before and after adding the acrylate layer reveals that the deformation from the LCE is restrained (Figure 2g and Figure S3). The amount of preserved actuation depends on the mechanical properties of the acrylate layer; for a composition without any dithiol, the layer is too stiff in the relevant temperature range to allow the LCE to fully deform, only reaching 66.5% of its original actuation capacity (Figure 2h and Figure S3). The maximum preserved actuation reaches a plateau around 90% for compositions with 10% monomer **2** or more, showing that the *T*_g_ is a major contributor to the actuation behavior, as compositions with the highest actuation have their *T*_g_ below the *T*_NI_ of the LCE. Specifically, for the 10% composition, the actuation below *T*_g_ at 35°C is suppressed (Figure 2g), narrowing the effective actuation window, which is an advantageous feature for haptic applications requiring controlled and repeatable switching. Therefore, in this work, we continue to use the 10% monomer **2** composition for our bilayer to form thermally addressable protrusions, which constitute the tactile pixels of our platform and can be selectively actuated to render tactile graphics.

The durability of the bilayer system was characterized by exposing the haptic material to 200 actuation cycles by heating and cooling the material between 20°C and 50°C. Comparing the 2D profiles of the first and 200th cycle shows no degradation in performance (Figure 2i). Furthermore, we extracted the deformation amplitude in the 50, 100, and 200th cycle and the results show no degradation compared with the initial state, see Figure S4. Figure 2j presents typical samples patterned with preprogrammed tactile icons using different stamp geometries, illustrating the shape versatility of the mechanical programming approach and its applicability to generate diverse tactile symbol sets. To provide more nuanced tactile information, the tendency of LCE actuation to occur across a temperature range rather than as an abrupt first‐order transition can be leveraged to control the protrusion height through precise temperature regulation (Figure S5). We also quantified the mechanical stiffness of the LCE layer via nanoindentation measurements (Figure S6), and the force generated upon actuation (Figure S7), providing parameters relevant to tactile perception [34].

### 2.2 Relationship Between Topological Features and Mechanical Programming During Embossing and Thermal Actuation

Considering the tactile parameters of our LCE haptic pixels, understanding the relationship between topological features and mechanical programming during embossing and thermal actuation is crucial for accurately designing the deformation of our LCE active coatings [35]. The mechanical programming techniques we use in our work are inspired by shape‐programming methodologies which are widely employed to inscribe complex three‐dimensional (3D) director configurations into LC materials [22, 23, 28, 36]; however, the topological–mechanical coupling mechanisms, which govern director‐field formation during embossing, remain underdefined. Therefore, we investigate how externally applied mechanical forces generate spatially varying orientational order in LCs by combining polarized optical microscopy (POM) with theoretical modeling. To validate our models, we measure the actuation of mechanically programmed topological structures with thermal stimuli.

POM imaging reveals the emergence of an azimuthal +1 topological defect surrounding the stamped indentation (Figure 3a and Figure S8), a feature independently confirmed by PolScope measurements (Figure 3b). Both techniques provide 2D projections of in‐plane mesogen alignment. To probe the director configuration through the film thickness, cross‐sectional analysis of an indented region was performed with POM imaging (Figure 3c and Figure S9), revealing a complex 3D orientational structure established during the embossing process (Figure 1(ci)). These observations indicate that localized mechanical deformation generates an organized director field extending beyond the immediate embossing region; based on this, two distinct deformation domains can be identified. The first domain is located directly beneath the embossed area and, consistent with the behavior of main‐chain elastomers, the long axes of the LC mesogens align preferentially along the direction of tensile strain, thereby orienting orthogonally to the compressive force imposed by the stamp. This deformation‐induced alignment produces a predominantly radial in‐plane configuration at the top surface (Figure 3e and Figure S10). The second domain extends laterally beyond the perimeter of the embossed region and exhibits a concentric director configuration (Figure 3e), implying a π/2 rotation of the orientational field across the boundary separating the embossed and non‐embossed deformation zones.

To rationalize the emergence of this nontrivial topology, we numerically computed the evolution of LC alignment using the neo‐classical theory of rubber elasticity adapted for LCEs (see Supporting Information for theoretical formulation and numerical implementation). The simulation geometry replicates the experimental configuration: the bottom surface of the LCE film is constrained in the xy‐plane at *z* = 0, while the top surface remains free except where in contact with the stamping tool. As shown in Figure 3d, compression by the stamp applied to an initially isotropic network (Figure 1(ci, ii)) generates a spatially heterogeneous stress field that decays through the film thickness, owing to the finite thickness of the elastomer, typically in the order of hundreds of µm. The resulting stress distribution induces a corresponding strain field, locally transforming the initially isotropic state into an anisotropic configuration. Yet, the interplay between the free upper boundary, the semi‐constrained lower boundary, and the curved deformation interface produces a complex 3D strain profile; analysis of the strain tensor components reveals coexistence of local compression and dilation (Figure S10), accompanied by significant strain gradients. On the free‐moving top surface, high compression within the embossed region generates radially distributed tensile strains, which drive formation of the mechanically induced radial director field. The maximum orientational order parameter (*S* ≈ 0.7) is located centrally beneath the indentation (Figure 3e).

Crucially, even within the embossed domain, the normal strain components *ε*_xx_ and *ε*_yy_ exhibit a sign inversion along the *z*‐axis (Figure S10). Specifically, these strains transition from positive, indicating extension, to negative, indicating contraction, outside the embossed region and vice versa within the embossed region as the bottom surface is approached. This spatial inversion results in a progressive transformation of the director configuration from a highly ordered radial pattern near the top surface to a weakly ordered azimuthal pattern near the bottom, consistent with the principal directions of the deformation gradient tensor. Outside the embossed area, material incompressibility drives lateral flow and downward displacement toward the indentation center (Figure 3f), producing compressive strains that give rise to the azimuthal deformation field. The superposition of radial alignment at the top surface and azimuthal alignment toward the bottom yields a bend–twist alignment profile across the thickness of the LCE active coating (Figure 3f). Simulations derived from the calculated director field match experimental POM observations (Figure 3a,c), validating our hypothesized alignment profile. Furthermore, in the embossed geometry, actuation manifests as flattening of the surface indentation, confirming our experimental observations; this phenomenon is further described analytically within our theoretical framework (Supporting Information).

### 2.3 Digital Device Integration of Liquid Crystal Elastomer Active Coatings

To harness our understanding and control over LCE haptic pixels, we integrate them in a digitally addressable tactile display. Fundamentally, the LCE active coating must be locally and independently actuated at spatial dimensions commensurate with individually programmed tactile pixels. To achieve this with thermal stimuli, we apply programmed LCE active coating material with a 2 mm pixel pitch on a thin printed circuit board (PCB), consisting of a matching square grid of surface‐mounted resistors, also spaced 2 mm apart (Figure 4a and Figure S11). We introduce thermally conductive paste at the interface of the LCE active coating and the PCB to enhance heat transfer, eliminate air gaps, and provide mechanical adhesion between the active coating and the electronic substrate. We then mount the PCB–LCE unit onto a heatsink for passive heat dissipation, which suppresses lateral thermal diffusion within the elastomer layer, thereby confining temperature increases to the vicinity of the activated resistor. Upon application of voltage within the PCB unit, Joule heating within a selected resistor generates a localized temperature rise in the overlying LCE region (Figure 4b,c), triggering actuation of the corresponding tactile pixel.

**FIGURE 4:**
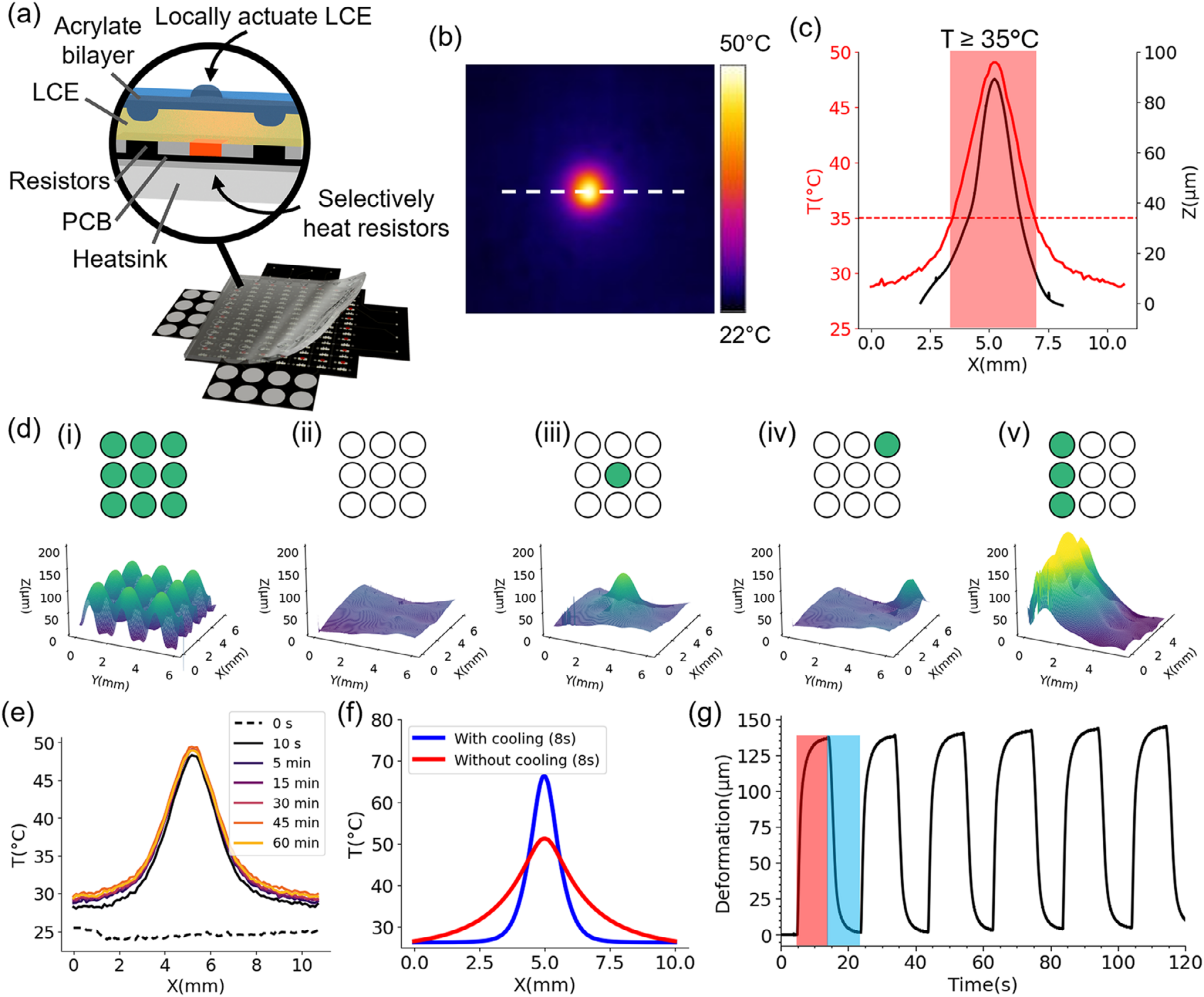
(a) Schematic of the demonstration setup, where the LCE‐based tactile material is placed on a thin flex PCB with a grid of resistors that can be individually heated via Joule heating. The red resistor represents the local heating, actuating one tactile pixel without affecting neighboring pixels. (b) Thermal image of the tactile material on the demonstration setup when a single resistor is heated. (c) Temperature profile on the surface of the material above the heated resistor (red), and height profile of a tactile pixel directly on top of the heated resistor (black). The area above 35°C is highlighted in red to emphasize that the lateral spread of heat overlaps with the actuation of the tactile pixel. (d) 3D profiles of a 3 × 3 grid of tactile pixels (i) at room temperature, (ii) when heating all pixels, (iii) when heating only the central pixel, (iv) when heating a single corner pixel, (v) when heating a row of pixels. (e) Temperature profile on the surface of the material above a heated resistor when heating for up to 1 h. (f) Simulated temperature profile on top of the LCE layer (below the acrylate bilayer) after locally heating for 8 s with (blue) and without (red) passive cooling in the form of a metal heatsink. After this time, an average temperature of 45°C was reached in a 2 mm × 2 mm area of the LCE. (g) Height profile of a pixel on the demonstration setup over time, heating a resistor (indicated in red in the first cycle) rapidly actuates a pixel, turning off the heating (indicated in blue) quickly reverts the actuation, returning the material to its flat state.

To understand the operation and control of our proof‐of‐concept device, we evaluate the spatial selectivity of our LCE pixels using a 3 × 3 pixel array. Uniform heating of the entire sample to 45°C induced simultaneous protrusion of all pixels (Figure 4d(i)), whereas in the absence of heating, the surface remained flat (Figure 4d(ii)). When voltage is applied exclusively to the central resistor, only the corresponding pixel actuates, while the surrounding eight pixels remain inactivated (Figure 4d(iii)). Similarly, selective activation of a corner resistor results in protrusion of a single corner pixel without inducing actuation in adjacent elements, including in previously activated sites (Figure 4d(iv)). Even under elevated power (>50°C), sufficient to produce amplified deformation in a row of three pixels, neighboring pixels outside the heated region remain flat (Figure 4d(v)). To investigate the temporal stability of this system, one resistor was continuously heated for 1 h (Figure 4e). The periodically recorded thermal profiles show a rise in background temperature of around 2°C around the pixel, however, the width of the region above the actuation temperature remains constant. The thermal images from which these profiles were extracted are shown in Figure S12. The essential role of heat dissipation in achieving this local heating profile was confirmed by simulating the system using finite element modeling with and without a passive cooling element in the form of a metal heatsink (Figure S13). After 8 s of heating, an average temperature above 45°C was reached in a volume of 2 × 2 × 0.5 mm of the LCE, representing the region responsible for actuation (Figure S14a). Figure 4f shows the resulting temperature profile on the top surface of the LCE (below the acylate layer), which has a much wider distribution in the absence of a heatsink compared to the narrow distribution with a heatsink. Increasing the heating time enlarges this difference (Figure S14b,c). Observing the heat dissipation in a 2D cross section of the material visualizes the temperature gradient laterally and through thickness (Figure S14d,e, Videos S1 and S2). Details on the energy requirements and efficiency are further explored in the Supporting Information.

We also quantify the actuation kinetics using a profilometer operated in static mode, monitoring the vertical displacement of a single pixel as a function of time. Figure 4g presents six consecutive heating‐cooling cycles, demonstrating reproducible and stable switching behavior over repeated operation. Upon activation, the pixel reaches a maximum protrusion height of 138 µm within 9.5 s, with 90% of the full displacement achieved in approximately 3 s. Following termination of power input, the structure relaxed to its flat state within 5 s, yielding a total on/off cycle time of approximately 15 s. Videos demonstrating the heating and cooling behavior with a passive heatsink and with active cooling are included in Videos S3 and S4, respectively. Although this response is slower than piezoelectrically actuated pin arrays, which typically operate on sub‐second timescales, a human user requires a few seconds to process graphical content via touch, and thus would not be inhibited by the activation time of the LCE pixels [37]. Moreover, modulation of the applied voltage enables continuous tuning of resistor temperature and, correspondingly, control of protrusion height (Figure S15).

These results confirm effective thermal confinement and demonstrate that the integrated PCB–LCE architecture enables independent, digitally addressable control of individual tactile pixels, suitable for use in digital devices and to provide a touch interface for the virtual world.

### 2.4 Liquid Crystal Elastomer Active Haptic Coatings in Real‐World Applications

We assess the suitability for real‐world applications of our concept through a standardized psychophysical test, namely a two‐alternative forced‐choice orientation discrimination task to determine perceptual thresholds in terms of just noticeable differences (JNDs), using the LCE material and swell paper [38]. Across repeated trials, twelve participants were sequentially presented with two‐line orientations rendered by our LCE pixels or on swell paper and asked to indicate whether the second stimulus was rotated clockwise or counterclockwise relative to the first; this set‐up is depicted in Figure 5a. By quantifying perceptual performance with this material and comparing that with performance with swell paper, we take a first step in testing whether this material is suitable to convey graphical information through touch.

**FIGURE 5:**
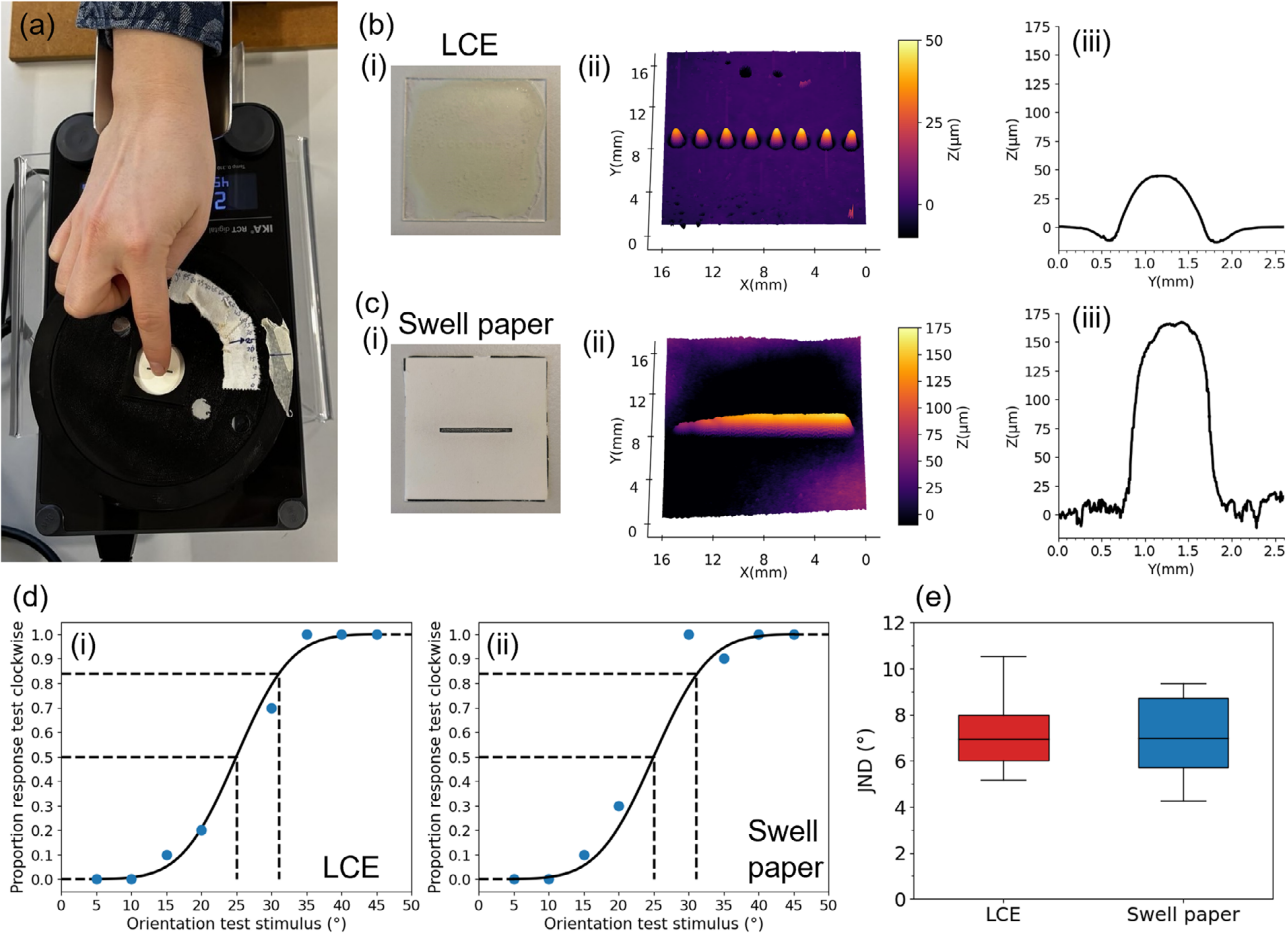
(a) Experimental set‐up for the perception study. An arm holder was used to keep the arm in a steady and equal position for all participants. The orientation of the stimuli was changed by the experimenter using a protractor. (b) (i) Picture of the LCE sample with bilayer (LCE layer with passive top‐coating) used for the perception study. (ii) The sample presents a line of raised dots when heated to 45°C. (iii) 2D profile of one of the pixels of the sample, showing that the protrusions have a height of 50 µm. (c) (i) Picture of the swell paper sample used for the perception study. (ii) 3D profile of the swell paper line, with similar dimensions in x and y to the dotted line of the LCE sample, but greater height. (iii) 2D profile of the swell paper line, showing a height of 170 µm. (d) Psychometric curves fitted over the proportion responses that the test was rotated clockwise compared to the reference of one participant with the LCE material (i) and swell paper (ii). The JND's were calculated by taking the absolute difference in orientation of the test stimulus where the participant indicated 84% and 50% of the time that the test stimulus was turned clockwise compared to the reference stimulus. This is indicated by the dotted lines in the figure. On the *x*‐axis the orientation of the test stimulus in degrees is shown. On the *y*‐axis the proportion that the participant responded that the test stimulus was turned clockwise compared to the reference stimulus. (e) Box plots of the JND's for all participants for both materials.

Swell paper is a widely adopted substrate for tactile graphics in educational materials and tactile maps for individuals with visual impairments. Swell paper is a thermally responsive paper that expands in regions printed with black ink upon exposure to heat, generating raised relief features that can be perceived through touch [39]; profilometer analyses of both LCE and swell paper surface features, shown in Figure 5b,c, are visually comparable. We chose a height of 50 µm for the LCE pixels because this is well above the detection threshold [40], and also well within the range of what is achievable with the material. Representative psychometric functions for one participant are shown in Figure 5d for both material conditions; the fitted curves exhibit comparable slopes. Figure 5e summarizes JND values across all participants, using box plot representations for each material type; visual inspection reveals closely overlapping distributions which indicate comparable performance. Statistical comparison using a paired‐samples t‐test confirmed that the mean JND for the LCE condition (*M* = 7.2°, SD = 1.6°) did not differ significantly from that of swell paper (*M* = 7.1°, SD = 1.7°), *t*(11) = −0.21, *p* = .84. To further substantiate this trend, we conducted an equivalence test with predefined bounds of −7.1° to 7.1° (*α* = 0.05). These equivalence bounds were chosen because the mean JND for the swell paper was 7.1°. This means that, according to our data, smaller orientation differences are not noticeable. Therefore, an effect of smaller orientation differences would not matter. The equivalence test yielded a significant result, *t*(11) = 10.8, *p* < 0.001. This shows that, within the equivalence bounds set, the orientation discrimination is comparable with both materials. Our evaluation demonstrates that tactile orientation discrimination performance achieved with LCE‐based protrusions is statistically comparable to that obtained using swell paper. This is a promising first step in determining whether the LCE material is a suitable material for tactile displays.

## 3 Conclusion

In our work, we developed mechanically programmed LCE coatings as a stimuli‐responsive digital platform for refreshable tactile displays; psychophysical benchmarking demonstrated that tactile line orientation discrimination performance of our LCEs is comparable to performance with swell paper, validating perceptual functionality alongside mechanical performance. This was achieved through mixture composition, two‐step cross‐linking, and embossing strategies with bilayer integration for our LCEs, enabling high‐amplitude protrusions (∼100 µm) within an operating window safe for human users. We further defined this behavior through simulations and used this understanding to successfully apply our LCE haptic coatings on a digitally addressable printed circuit board; spatially confined Joule heating was achieved with patterned resistors, capable of repeatably switching LCE pixels in the order of seconds as well as controlling their height. We envision our proceeding work will connect the haptic functions of our device with commonly used human–machine interfaces, including displays, virtual/augmented/mixed reality devices and potentially even surgical robotic controllers. Hence, our work establishes LCE coatings as a scalable and perceptually viable material to enable the next‐generation dynamic tactile interfaces for an accessible and equal virtual world.

## 4 Experimental Section

### 4.1 LCE Preparation

The monomers used to prepare the LCE film are depicted in Figure 1b. The diacrylate mesogen monomer **1** (C6BAPE (4‐(6‐(acryloyloxy)hexyloxy)phenyl‐4‐(6‐(acryloyloxy)hexyloxy) benzoate), Daken Chemical Limited), dithiol monomer **2** (DODT (2,2′‐(Ethylenedioxy)diethanethiol), Merck Life Science NV), tetrathiol monomer **3** (PETMP (Pentaerythritol tetrakis(3‐mercaptopropionate), Merck Life Science NV), divinyl monomer **4** (TEGDE (triethylene glycol divinyl ether), Merck Life Science NV), tetravinyl monomer **5** (GDA (glyoxal bis(diallyl acetal), Merck Life Science NV) photoinitiator Irgacure 184 (1‐hydroxy‐cyclohexyl‐phenyl‐ketone, Basf), and radical scavenger BHT (2,6‐di‐*tert*‐butyl‐4‐methylphenol, Merck Life Science NV) were first dissolved in DCM (dichloromethane) in a concentration of 566.7 mg ml^−1^ and mixed using a magnetic stirrer at 400–600 rpm. A catalytic amount (0.2 wt% with respect to the total weight of the reaction mixture, not including solvent) of the base DPA (dipropylamine, Merck Life Science NV) was added to catalyze the Michael addition reaction between the thiol and acrylate groups. After mixing for 10 s, the solution was pipetted onto 3 × 3 cm glass substrates with 0.5 mL per sample and left to evaporate the solvent and react for at least 5 h. The resulting coatings were then dried in vacuum at 50°C for another minimum 5 h to fully remove the DCM. The compositions of the LCE mixtures are given in Table 1:

**TABLE 1:** Compositions in wt%. of the LCE's used in this research.

	Mixtures (wt.%) (named after molar percentage of vinyl groups from 5)				
Names	**0%**	**10%**	**20%**	**33%**	**100%**
C6BAPE (monomer 1)	62.6	62.7	62.9	63	63.7
DODT (monomer 2)	16.6	16.6	16.7	16.7	16.9
PETMP (monomer 3)	13.6	13.6	13.7	13.7	13.9
TEGDE (monomer 4)	4.5	4.1	3.6	3.0	0
GDA (monomer 5)	0	0.3	0.6	0.9	2.9
Irgacure 184 (photoinitiator)	2	2	2	2	2
BHT (radical inhibitor)	0.6	0.6	0.6	0.6	0.6

To program the surface topography, samples were first cooled to 5°C on a homemade Peltier‐element‐based cooling stage to ensure that the nematic phase is maintained during the shape programming and photopolymerization. A 3D printed stamp (printed using a Formlabs Form 3 with Grey V4 resin) was then placed on the coating and weighed down by placing a 7.8 kg weight on the stamp. After 5 min, the stamp was removed and a nitrogen atmosphere was established around the sample. The sample was then exposed to UV light using an Excelitas Technologies OMNICURE SERIES 2000 UV source at an intensity of 20 mW cm^−2^ for 30 min. The sample was flipped halfway through the illumination time to ensure even curing throughout the sample thickness.

### 4.2 Bilayer Preparation

The bilayer was prepared by mixing varying ratios of monomer **2**, monomer **6** (tripropylene glycol diacrylate (TPGDA), Merck Life Science NV), and Irgacure 184 until homogeneous. A few drops of this mixture were pipetted onto a fully cross‐linked LCE film and a glass plate was placed on top to flatten out the TPGDA mixture. A nitrogen atmosphere was established and the sample was exposed to 10 mW cm^−2^ light for 5 min. Finally, the glass plate was separated from the film to reveal the flat bilayer. The compositions of the mixtures are given in Table 2:

**TABLE 2:** Compositions in wt%. of the acrylate layers used in this research.

	Mixtures (wt.%) (named after wt.% of dithiol monomer 2 dissolved in diacrylate monomer 6)				
Name	**0%**	**5%**	**10%**	**15%**	**20%**
TPGDA (monomer 6)	98	93	88	83	78
DODT (monomer 2)	0	5	10	15	20
Irgacure 184 (photoinitiator)	2	2	2	2	2

### 4.3 Characterization of the Tactile Film and Its Thermal Response

The 3D surface topographies of the produced materials at different temperatures were studied using a Sensofar S Neox in interferometry mode using a 5x objective. The swell paper used in the perceptual experiment was characterized in the same way. The dynamic actuation measurements were measured using a Bruker Dektak XT in static mode. The actuation force was measured using a Novatech 20 N Loadcell. The LCE film and the acrylate bilayers mechanical properties were studied using dynamic mechanical thermal analysis (DMTA) using a TA instruments Q800 DMA, the measured LCE samples were fully cross‐linked in a polydomain state without shape programming. The films were placed in tensile clamps and oscillated with an amplitude of 15 µm at 1 Hz while heating from −40°C to 100°C at a rate of 10°C min^−1^. The indentation modulus of the material was measured using an MTS Nanoindenter XP with a steel spherical tip with a radius of 250 µm. The thermal capacities of the LCE and acrylate layer that were used for thermal simulations and energy calculations were measured using a TA instruments DSC 2500 with hermetic aluminium pans. The cyclic heating and cooling to test the durability of the bilayer material was performed using a TA instruments DSC 250. One pixel was cut from a sample, placed in an aluminium DSC pan without a lid and repeatedly heated and cooled at a rate of 10°C min^−1^ between 20°C and 50°C. Each time these target temperatures were reached, the sample was held isothermal for 5 min before continuing the cycles. The surface topographies before and after these heating cycles were characterized using interferometry. A Leica DM6000 polarized optical microscope (POM) was used to characterize the alignment around the indents in the LCE.

### 4.4 Polscope

The birefringence and director field of the LCE samples were measured using a commercial birefringence imaging system (LCC7201B, Thorlabs Inc.).

### 4.5 Simulations

We employ neo‐classical theory for LCEs coupled with the nematic free energy to analyze the mechanically induced and temperature‐variation‐induced orientational field and LCE deformation. The description of the theories and numerical implementation can be found in Supporting Information.

### 4.6 Device Integration

Chip resistors (RC0100FR‐0710RP YAGEO), mounted on a flexible PCB were used to actuate the LCE through Joule heating. The PCB was placed on top of a 50 × 50 × 4 mm Peltier element, which was used as a passive heatsink after realizing that it was not necessary to actively cool the material.

### 4.7 Finite Element Modeling (FEM) analysis

COMSOL Multiphysics (version 6.1) was used to simulate the electro‐thermal behavior of the LCE‐based haptic actuator. A 3D multi‐physics model was developed by coupling two physics modules: the Electric Currents interface and the Heat Transfer in solids interface, linked via the Joule Heating multi‐physics coupling. This approach enabled the calculation of ohmic heat generation within the resistive element and the resulting temperature distribution across the multilayer structure.

### 4.8 Perception Study

The perceptual experiment was done using a psychophysical method in a lab setting. The experiment was designed to measure discrimination thresholds for an orientation discrimination task. This was done using a two‐alternative forced choice (2AFC) method combined with a method of Constant Stimuli. Two types of stimuli were used:

1. A square shaped sample (3 by 3 cm) of the LCE with a dotted line with a length of 1.5 cm. The line consisted of 8 dots of approximately 50 µm high and with a diameter of 1.2 mm.
2. A square shaped sample (3 by 3 cm) of swell paper with a line with a length of 1.5 cm. The line was approximately 175 µm high and 1.2 mm thick.

Both stimuli were presented on a heated plate set to 45°C. The stimuli were placed in a 3D‐printed stimulus holder. The orientation of the stimuli was controlled manually, by rotating the stimuli on a protractor in the horizontal field.

Prior to the experiment, participants signed the informed consent form. Participants (*N* = 12, mean age = 28, all right‐handed, seven females) were sequentially presented with two orientations of a stimulus. One of the two orientations was the reference orientation, which was oriented at 25°. The other orientation was the test orientation. This was either 5°, 10°, 15°, 20°, 30°, 35°, 40°, or 45°. Every combination of test and reference orientation was presented 10 times. Five times, the reference orientation was presented first, and the other 5 times the test orientation was presented first. The order of the orientations of the test stimulus was block randomized.

Participants were asked to indicate whether the second line orientation was rotated clockwise or counterclockwise compared to the first line orientation. The proportion of trials in which the participants indicated that the test orientation was turned clockwise compared to the reference orientation (*y*‐axis) was plotted against the orientation of the test stimulus (*x*‐axis). A cumulative Gaussian was fitted to the data points using MATLAB with the psignifit toolbox [41]. This was done for each participant and both material types separately. We calculated the JND by taking the absolute difference in orientation at the points where the participant indicated 84% and 50% of the trials that the test stimulus was turned clockwise compared to the reference stimulus [38].

The JND represents the discrimination threshold. This is the minimum orientation difference that participants can reliably detect. The mean JND was then calculated for each material type over all participants. A paired t‐test was done to determine whether there was a significant difference between the JNDs of the two materials. Additionally, an equivalence test was done to investigate whether line orientation discrimination performance of the two materials was indeed comparable within set equivalence bounds [42]. We tested normality of the distribution for the differences between the JND for the condition with LCE material distances and the condition with swell paper using a Shapiro–Wilk test. Normality was assumed (*W* = 0.96, *p* = 0.74).

This experiment was approved by the Ethical Review Board of the Human‐Technology Interaction group from Eindhoven University of Technology.

## Funding

This research forms part of the research program financed by the Dutch Research Council (NWO) (OTP19440, OTP19966, and Gravity Program 024.005.020 – Interactive Polymer Materials IPM) as well as the Natural Science Foundation of Guangdong Province (no. 2024B1515040023), the GJYC program of Guangzhou (no. 2024D03J0002), and the TCL Young Talents program.

## Conflicts of Interest

The authors declare no conflicts of interest.

## Supporting information




**Supporting File 1**: adma74546‐sup‐0001‐SuppMat.docx.

**Supporting File 2**: adma74546‐sup‐0002‐VideoS1‐S4.zip.

## Data Availability

The data that support the findings of this study are available from the corresponding author upon reasonable request.

## References

[1] I. Levin and D. Mamlok , “Culture and Society in the Digital Age,” Information 12 (2021): 68, 10.3390/INFO12020068.

[2] S. Hamideh Kerdar , L. Bächler , and B. M. Kirchhoff , “The Accessibility of Digital Technologies for People with Visual Impairment and Blindness: A Scoping Review,” Discover Computing 27, no. 24 (2024), 10.1007/S10791-024-09460-7.

[3] Y. Shi and G. Shen , “Haptic Sensing and Feedback Techniques Toward Virtual Reality,” Research 7 (2024): 0333, 10.34133/RESEARCH.0333. PMC10964227 38533183

[4] C. Bermejo and P. Hui , “A Survey on Haptic Technologies for Mobile Augmented Reality,” ACM Computing Surveys (CSUR 54 (2021): 1–35, 10.1145/3465396.

[5] M. Hamash , H. Ghreir , and P. Tiernan , “Breaking Through Barriers: A Systematic Review of Extended Reality in Education for the Visually Impaired,” Education Sciences 14 (2024): 365, 10.3390/EDUCSCI14040365.

[6] P. Edman , Tactile Graphics, American Foundation for the Blind, New York, NY (1992).

[7] T. Bhatnagar , A. Higgins , N. Marquardt , M. Miodownik , and C. Holloway , “Analysis of Product Architectures of Pin Array Technologies for Tactile Displays,” Proceedings of the ACM on Human‐Computer Interaction 7 (2023): 135–155, 10.1145/3626468.

[8] D. Leonardis , L. Claudio , and A. Frisoli , “A Survey on Innovative Refreshable Braille Display Technologies,” Advances in Intelligent Systems and Computing 800 (2017): 488–498, 10.1007/978-3-319-60597-5_46.

[9] W. Yang , J. Huang , R. Wang , W. Zhang , H. Liu , and J. Xiao , “A Survey on Tactile Displays for Visually Impaired People,” IEEE Transactions on Haptics 14 (2021): 712–721, 10.1109/TOH.2021.3085915. 34077370

[10] L. Zeng and G. Weber , “The Overview of Haptic Displays,” in Advancements in Pin‐Array Tactile Displays (2025), 15–28, 10.1007/978-3-032-03151-8_2.

[11] T. J. White and D. J. Broer , “Programmable and Adaptive Mechanics With Liquid Crystal Polymer Networks and Elastomers,” Nature Materials 14 (2015): 1087–1098, 10.1038/nmat4433. 26490216

[12] P. Lv , X. Yang , H. K. Bisoyi , et al., “Stimulus‐Driven Liquid Metal and Liquid Crystal Network Actuators for Programmable Soft Robotics,” Materials Horizons 8 (2021): 2475–2484, 10.1039/D1MH00623A. 34870302

[13] X. Zhang , J. Wei , L. Qin , and Y. Yu , “Liquid Crystal Polymer Actuators With Complex and Multiple Actuations,” Journal of Materials Chemistry B 12 (2024): 6757–6773, 10.1039/D4TB01055H. 38916076

[14] J. Hu , M. Yu , M. Wang , K.‐L. Choy , and H. Yu , “Design, Regulation, and Applications of Soft Actuators Based on Liquid‐Crystalline Polymers and Their Composites,” ACS Applied Materials & Interfaces 14 (2022): 12951–12963, 10.1021/acsami.1c25103. 35259869

[15] K. M. Herbert , H. E. Fowler , J. M. McCracken , K. R. Schlafmann , J. A. Koch , and T. J. White , “Synthesis and Alignment of Liquid Crystalline Elastomers,” Nature Reviews Materials 7 (2021): 23–38, 10.1038/s41578-021-00359-z.

[16] N. A. Traugutt , R. H. Volpe , M. S. Bollinger , et al., “Liquid‐Crystal Order During Synthesis Affects Main‐Chain Liquid‐Crystal Elastomer Behavior,” Soft Matter 13 (2017): 7013–7025, 10.1039/c7sm01405h. 28930352

[17] A. Gablier and E. M. Terentjev , “Flexible Force‐Bearing Liquid Crystalline Elastomer Component Toward a Dynamic Braille Platform,” Nano Select 4 (2023): 324–332, 10.1002/nano.202300026.

[18] N. Torras , K. E. Zinoviev , J. Esteve , and A. Sánchez‐Ferrer , “Liquid‐Crystalline Elastomer Micropillar Array for Haptic Actuation,” Journal of Materials Chemistry C 1 (2013): 5183, 10.1039/c3tc31109k.

[19] C. J. Camargo , H. Campanella , J. E. Marshall , et al., “Batch Fabrication of Optical Actuators Using Nanotube–Elastomer Composites Towards Refreshable Braille Displays,” Journal of Micromechanics and Microengineering (2012): 075009, 10.1088/0960-1317/22/7/075009.

[20] N. Torras , K. E. Zinoviev , C. J. Camargo , et al., “Tactile Device Based on Opto‐Mechanical Actuation of Liquid Crystal Elastomers,” Sensors and Actuators A: Physical 208 (2014): 104–112, 10.1016/j.sna.2014.01.012.

[21] X. Dong , Y. Jiang , Y. Xu , et al., “Multisensory Flexible Braille Interactive Device Based on Liquid Crystal Elastomers,” ACS Applied Electronic Materials 4 (2022): 3834–3840, 10.1021/acsaelm.2c00458.

[22] C. M. Yakacki , M. Saed , D. P. Nair , et al., “Tailorable and Programmable Liquid‐Crystalline Elastomers Using a Two‐Stage Thiol–Acrylate Reaction,” RSC Advances 5 (2015): 18997–19001, 10.1039/C5RA01039J.

[23] M. Barnes and R. Verduzco , “Direct Shape Programming of Liquid Crystal Elastomers,” Soft Matter 15 (2019): 870–879, 10.1039/c8sm02174k. 30628627

[24] P. Lyu and D. Liu , “Liquid Crystal Elastomer Soft Robotic Arm for Pick‐and‐Place Operation Controlled by Light,” Proceedings of the SPIE 12907 (2024): 1290704, 10.1117/12.3000162.

[25] Q. He , Z. Wang , Y. Wang , A. Minori , M. T. Tolley , and S. Cai , “Electrically Controlled Liquid Crystal Elastomer–Based Soft Tubular Actuator With Multimodal Actuation,” Science Advances 5 (2019): eaax5746, 10.1126/sciadv.aax5746. PMC6788870 31646178

[26] C. Yuan , D. J. Roach , C. K. Dunn , et al., “3D Printed Reversible Shape Changing Soft Actuators Assisted by Liquid Crystal Elastomers,” Soft Matter 13 (2017): 5558–5568, 10.1039/C7SM00759K. 28721407

[27] S. A. M. Weima , R. Norouzikudiani , J. Baek , et al., “Human Interactive Liquid Crystal Fiber Arrays,” Science Advances 10 (2024): eadp0421, 10.1126/sciadv.adp0421. PMC11378903 39241076

[28] J. Küpfer and H. Finkelmann , “Nematic Liquid Single Crystal Elastomers,” Die Makromolekulare Chemie, Rapid Communications 12 (1991): 717–726, 10.1002/MARC.1991.030121211.

[29] M. O. Saed , C. P. Ambulo , H. Kim , et al., “Molecularly‐Engineered, 4D‐Printed Liquid Crystal Elastomer Actuators,” Advanced Functional Materials 29 (2019): 1806412, 10.1002/ADFM.201806412.

[30] B. B. Ong and N. Milne , “INJURY, *FATAL and NONFATAL | Burns and Scalds* ,” Encyclopedia of Forensic and Legal Medicin** *e* ** (2005): 173–181, 10.1016/B978-0-12-800034-2.00220-2.

[31] D. Wang , H. Zhang , E. Arens , and C. Huizenga , “Observations of Upper‐Extremity Skin Temperature and Corresponding Overall‐Body Thermal Sensations and Comfort,” Building and Environment 42 (2007): 3933–3943.

[32] W. Redisch , E. Sheckman , and J. M. Steele , “Skin Temperature Response of Normal Human Subjects to Various Conditions,” Circulation 6 (1952): 862–867, 10.1161/01.CIR.6.6.862. 12998111

[33] T. S. Hebner , H. E. Fowler , K. M. Herbert , N. P. Skillin , C. N. Bowman , and T. J. White , “Polymer Network Structure, Properties, and Formation of Liquid Crystalline Elastomers Prepared via Thiol–Acrylate Chain Transfer Reactions,” Macromolecules 54 (2021): 11074–11082, 10.1021/ACS.MACROMOL.1C01919.

[34] T. A. Kern , Actuator Design, in Engineering Haptic Devices (Springer, 2023), 309–429, 10.1007/978-3-031-04536-3.

[35] M. Barnes and J. S. Biggins , “Microstructural Basis of Complex Mechanical Programming in Liquid Crystal Elastomers,” Journal of Elasticity 157 (2025): 48, 10.1007/S10659-025-10138-4. PMC12122613 40453202

[36] T. Bruining , D. R. Tomé , and D. Liu , “Shape Programming of Liquid Crystal Elastomers by Two‐Stage Wavelength‐Selective Photopolymerization,” Materials Horizons 13 (2026): 243–251, 10.1039/D5MH01907A. PMC12582633 41183367

[37] M. I. H. Shamsul Bahrin , H. Md Yusof , S. N. Sidek , and A. S. Ghazali , “An Early Investigation Into Raised‐Line Tactile Graphics Reading Behavior Among Blind and Visually Impaired Individuals,” Jurnal Kejuruteraan 36 (2024): 1103–1125, 10.17576/jkukm-2024-36(3)-22.

[38] B. A. Frederick and A. L. Cecala , “Book Review Psychophysics: A Practical Introduction (2nd Ed.),” Journal of Undergraduate Neuroscience Education 15 (2016): R21–R21.

[39] T. Cheng , “SwellSense: Creating 2.5D Interactions With Micro‐Capsule Paper,” in Conference on Human Factors in Computing Systems—Proceedings (2023): 1–13, 10.1145/3544548.3581125.

[40] R. S. Johansson and R. H. Lamotte , “Tactile Detection Thresholds for a Single Asperity on an Otherwise Smooth Surface,” Somatosensory Research 1 (1983): 21–31, 10.3109/07367228309144538. 6679912

[41] F. A. Wichmann and N. J. Hill , “The Psychometric Function: I. Fitting, Sampling, and Goodness of Fit,” Perception & Psychophysics 63 (2001): 1293–1313, 10.3758/BF03194544/METRICS. 11800458

[42] D. Lakens , A. M. Scheel , and P. M. Isager , “Equivalence Testing for Psychological Research: A Tutorial,” Advances in Methods and Practices in Psychological Science 1 (2018): 259–269, 10.1177/2515245918770963.

