# Supplementary material for: Liquid Crystal Elastomer‐Based Haptic Pixel Arrays at Your Fingertips for Advanced Human–Machine Interfaces
Source: Adv Mater. 2026 Aug 9;38(56):e74546. doi: 10.1002/adma.74546 (PMC13648916; doi:10.1002/adma.74546)
Supplement: Supplementary file 1 [file ADMA-38-e74546-s002.docx]

Supporting Information

Liquid crystal elastomer based haptic pixel arrays at your fingertips for advanced human-machine interfaces

Tom Bruining, Enjun Lin, Yu Zou, Meike Heldoorn, Pengrong Lyu, Samuël A.M. Weima, Astrid M.L. Kappers, Myrthe A. Plaisier, Satoshi Aya*, Danqing Liu*

**Optimization of the actuation temperature range**

**Figure S1.** ((Actuation percentage (defined as the amount of actuation relative to the initial depth of the indentation in the LCE at room temperature) plotted over temperature for a range for compositions containing different percentages of vinyl groups from monomer **5**. The nematic to isotropic transition temperatures of the compositions were derived from this data as the points with the highest slope.))

**Dynamic mechanical analysis of LCE compositions**

**Figure S2.** ((Storage modulus, loss modulus and tan delta as measured using DMTA for the different LCE compositions used in this research.))

**Effect of varying bilayer compositions on actuation**

**Figure S3.** ((2D profiles of the surfaces of adjacent tactile pixels before and after adding acrylate bilayers with different compositions. The figures on the right show the actuation measured at the lowest point of the dent or highest point of the bump to visualize how much of the actuation capacity from the LCE is preserved after adding the bilayer.))

**Durability over numerous actuation cycles**

**Figure S4.** ((Deformation amplitude before cyclic actuation studies, and on the 50th, 100th and 200th actuation cycles. The sample used was produced 2 years and 3 months prior to performing this cyclic actuation test, which further exemplifies the durability of the material.))

**Gradual actuation**

**Figure S5.** ((3D plots of the tactile pixels at different temperatures show that stable states are obtained with different levels of surface protrusion.))

**Indentation testing**

**Figure S6.** ((An example of indentation test results for the LCE layer at room temperature and 40 °C.))

To determine the indentation modulus of the LCE material, we performed nanoindentation tests. The material was indented using a steel tip with a radius of 250 µm to an indentation depth of 10 µm, a grid of 5x5 points were measured and an average was taken as the final value. The value for the indentation modulus was calculated using the following formulae:

$\frac{1}{E_{I}^{*}}=\frac{1-\nu^{2}}{E_{I}}+\frac{1-\nu_{ind}^{2}}{E_{ind}}$	(S1)

Where:

E^*^_I_ =Reduced modulus of the sample

E_I_ = Elastic modulus of the sample

ν = Poisson ratio of the sample (0.4)

ν_ind_ = Poisson ratio of the indenter (0.3)

E_ind_ = Elastic modulus of the indenter (210 GPa)

This equation can be rewritten to the following form to calculate the indentation modulus of the sample:

$E_{I}=\frac{(1-\nu^{2})E_{I}^{*}E_{ind}}{E_{ind}-E_{I}^{*}(1-\nu_{ind}^{2})}$	(S2)

$E_{I}^{*}=\frac{S\sqrt{\pi}}{2\sqrt{A_{p}}}$	(S3)

$S=\frac{dP}{dh}$	(S4)

$A_{p}=2\pi rh_{p}$	(S5)

r = Radius of the indentation tip (250 µm)

A_p_ = Projected contact area between the indenter and the sample

P = Load on the sample

S = The slope of the graph, taken at the end of the load segment

h_p_ = Indenter penetration depth

The average indentation modulus of the measured points is 3.81 MPa at room temperature and 2.48 MPa at 40 °C.

**Actuation force**

**Figure S7.** ((Force of actuation measured over time in a heating-cooling cycle of the haptic material.))

To characterize the force produced by the thermal actuation of the material, we placed a load cell in direct contact with the acrylate bilayer on tactile film in its flat state at room temperature. We then heated the sample to 45°C, after which the sample was cooled and relaxed to its flat state again. The measured force approaches 0.2 N, which is the recommended blocking force for piezo based tactile displays.

**Polarized optical microscopy (POM)**

**Figure S8.** ((POM images of the top view of a dent in the LCE film. When rotating the analyzer of the POM while observing a dent in the LCE film, the Maltese cross rotates in the same direction, indicating that the sign of the defect is positive.))

**Figure S9.** ((POM image of the cross section of a programmed dent in the LCE film at 50°C. At this temperature the dent has relaxed to its flat state.))

**Neo-Classical Theory for LCE**

The classical theoretical framework for describing LCE deformation is based on the assumption of an anisotropic Gaussian distribution of polymer chain conformations, which are constrained by crosslinking. This theory integrates mechanical deformation with nematic order and the director field. It is widely used to explain the relationship between the LCE deformation and the reorientation of the director field. In the theory, the mechanical elastic (ME) free energy density can be expressed as follows:

$$\begin{aligned} f_{\mathrm{ME}}=\frac{1}{2}\mu\mathrm{Tr}\left( {\underset{\boldsymbol{=}}{\mathcal{l}}}_{0}\cdot{{\underset{=}{\boldsymbol{\lambda}}}_{t}}^{T}\cdot{\underset{=}{\mathcal{l}}}^{-1}\cdot{\underset{=}{\boldsymbol{\lambda}}}_{t} \right)\#\left( 1 \right) \end{aligned}$$

$$\begin{aligned} \underset{=}{\mathcal{l}}=\mathcal{l}_{\perp} \underset{=}{\boldsymbol{\delta}}+\delta\mathcal{l}\mathbf{nn}\#\left( 2 \right) \end{aligned}$$

$$\begin{aligned} {\underset{=}{\boldsymbol{\lambda}}}_{t}=\boldsymbol{\delta}+\nabla\mathbf{u}\#\left( 3 \right) \end{aligned}$$

Here, $\mu$ denotes the rubber modulus. ${\underset{=}{\boldsymbol{\lambda}}}_{t}$ is the deformation gradient tensor, defined in Eq. (3). $\underset{=}{\mathcal{l}}$ is the effective step length tensor, which relates the director field $\mathbf{n}$ to the shape anisotropy $\delta\mathcal{l (}\delta\mathcal{l=}\mathcal{l}_{\parallel}-\mathcal{l}_{\perp})$ as given by Eq. (2). The initial step length tensor ${\underset{\boldsymbol{=}}{\mathcal{l}}}_{0}$ depicts the initial director field and is a fixed tensor, thereby capturing the memory effect characteristic of LCEs. The step length tensor $\underset{=}{\mathcal{l}}$ at a specific time *t* couples with the deformation gradient tensor ${\underset{=}{\boldsymbol{\lambda}}}_{t}$ in a manner that depends on the shape anisotropy $\delta\mathcal{l}$ of the polymer chains. If $\delta\mathcal{l=}0$, the polymer network becomes isotropic, and $\mathcal{l}$ reduces to $\underset{=}{\mathcal{l}}=\mathcal{l}_{\perp} \underset{=}{\boldsymbol{\delta}}$, corresponding to a spherical conformation of the polymer chains. This situation decouples the director $\mathbf{n}$ with the deformation gradient tensor ${\underset{=}{\boldsymbol{\lambda}}}_{t}$. If $\delta\mathcal{l\neq}0$, this anisotropy introduces a directional elastic response. Specifically, when $\delta\mathcal{l>}0$, corresponding to a prolate conformation, the director $\mathbf{n}$ tends to align along the directions of tensile deformation to minimize the anisotropic elastic free energy, while reorienting away from the compressive axes. Conversely, when $\delta\mathcal{l<}0$, corresponding to an oblate conformation, the director $\mathbf{n}$ prefers to align along compressive deformation and reorient away from the tensile axes. In our experiment, our materials belong to the class of main-chain elastomers, which corresponding to the prolate polymer chains ($\delta\mathcal{l>}0$).

Since the shape anisotropy $\delta\mathcal{l}$ is proportional to the nematic order parameter *s*, such that $\delta\mathcal{l/}\mathcal{l}_{\perp}\simeq\alpha S$, Eq. (2) can be reformulated as:

$$\begin{aligned} \underset{=}{\mathcal{l}}=\mathcal{l}_{\perp} \left( \underset{=}{\boldsymbol{\delta}}+\alpha s \mathbf{nn} \right),\#\left( 4 \right) \end{aligned}$$

where the coefficient $\alpha$ should be positive for the case of the prolate conformation.

**Total** **Free Energy of System**

In addition to the mechanical elastic free energy density $f_{\mathrm{ME}}$, Landau free energy $f_{\mathrm{Land}}$and elastic free energy in uniaxial nematic phase $f_{\mathrm{ela}}$ in the framework of $\mathbf{Q}$-tensor theory are considered:

$$\begin{aligned} f_{\mathrm{Land}}=\frac{A}{2} \mathrm{Tr}\left( \mathbf{Q}^{2} \right)+\frac{B}{4} \left[ \mathrm{Tr}\left( \mathbf{Q}^{2} \right) \right]^{2}-\frac{C}{\mu}\sigma_{v}\mathrm{Tr}\left( \mathbf{Q} \right)\#\left( 5 \right) \end{aligned}$$

$$\begin{aligned} f_{\mathrm{ela}}=\frac{L}{2}\frac{\partial Q_{ij}}{\partial x_{k}}\frac{\partial Q_{ij}}{\partial x_{k}}\#\left( 6 \right) \end{aligned}$$

$$\begin{aligned} \mathbf{Q}=s\left( \mathbf{n}\boldsymbol{\bigotimes}\mathbf{n}\boldsymbol{-}\frac{1}{3}\mathbf{I} \right)\boldsymbol{\#}\left( 7 \right) \end{aligned}$$

$$\begin{aligned} \sigma_{v}=\sqrt{\frac{3}{2}\left( \sigma_{ij}-\frac{1}{3}\delta_{ij}\sigma_{kk} \right)\left( \sigma_{ij}-\frac{1}{3}\delta_{ij}\sigma_{kk} \right)}\#\left( 8 \right) \end{aligned}$$

In Eq. (5), $A$ is a temperature-dependent coefficient, taking the form $A=A_{0}\left( T-T^{*} \right)$. $T^{*}$ denotes the isotropic–nematic transition temperature. With a positive coefficient $B$, the equation governs the thermodynamic stability of the nematic order and determines the equilibrium value of the order parameter $s^{*}$. The coefficient $C$ characterizes the coupling between the order parameter $s$ and equivalent mechanical stress $\sigma_{v}$ defined by Eq. (8). This term works to modify the equilibrium order parameter $s^{*}$ under an applied stress. Eq. (6) penalizes spatial variations in the director field, where the effective elastic constant $L$ characterizes the resistance to orientational distortions.

Overall, one has to sum up all the free energy to obtain the total free energy of the system:

$$\begin{aligned} f_{\mathrm{tot}}=f_{\mathrm{ME}}+f_{\mathrm{Land}}+f_{\mathrm{ela}}\#\left( 9 \right) \end{aligned}$$

**Lagrangian formulation: computation of LCE deformation through global stiffness matrix**

We employ the Lagrangian formulation to calculate the mechanical deformation of LCEs under external loading. The computational domain is discretized using linear tetrahedral elements (4 nodes, T4), with each node possessing three translational degrees of freedom ($x_{j}$; $j=1, 2, 3$). The constitutive matrix $\mathbf{C}$ is derived based on Eq. (9), and is calculated through the following equations:

$$\begin{aligned} \varepsilon_{\mathrm{ij}}=\frac{\partial u_{i}}{\partial x_{j}}\#\left( 10 \right) \end{aligned}$$

$$\begin{aligned} C_{\mathrm{ij}}=\frac{\partial^{2}f_{\mathrm{tot}}}{\partial\varepsilon_{\mathrm{ij}}^{2}}=\frac{\partial^{2}f_{\mathrm{ME}}}{\partial\varepsilon_{\mathrm{ij}}^{2}}\#\left( 11 \right) \end{aligned}$$

Then, we have to assemble element stiffness matrix $\mathbf{D}$ based on the constitutive matrix $\mathbf{C}$ and the T4 elements. For each T4 element, $N_{i}\left( \boldsymbol{\xi} \right)$ is the linear shape functions of the 4-node tetrahedral element with respect to the reference coordinates $\boldsymbol{\xi}$. Thus, the gradient of shape function can be defined by a constant matrix:

$$\begin{aligned} \frac{\partial\mathbf{N}}{\partial\boldsymbol{\xi}}\mathbf{=}\left[ \frac{\partial N_{1}}{\partial\boldsymbol{\xi}}\mathbf{,}\frac{\partial N_{2}}{\partial\boldsymbol{\xi}}\mathbf{,}\frac{\partial N_{3}}{\partial\boldsymbol{\xi}}\mathbf{,}\frac{\partial N_{4}}{\partial\boldsymbol{\xi}} \right]\mathbf{=}\left[ \begin{matrix} -1 & 1 & 0 & 0 \\ -1 & 0 & 1 & 0 \\ -1 & 0 & 0 & 1 \end{matrix} \right]\boldsymbol{\#}\left( 12 \right) \end{aligned}$$

Given the global coordinates of the four nodes in the element in Eq. (13), we can get the Jacobian matrix $\mathbf{J}$ as shown in Eq. (14), which maps reference coordinates to physical coordinates.

$$\begin{aligned} \mathbf{u}^{'}=\left[ \begin{aligned} \mathbf{u}_{\boldsymbol{1}} \\ \mathbf{u}_{\boldsymbol{2}} \\ \mathbf{u}_{\boldsymbol{3}} \\ \mathbf{u}_{\boldsymbol{4}} \end{aligned} \right]\boldsymbol{=}\left[ \begin{matrix} u_{1x} & u_{1y} & u_{1z} \\ u_{2x} & u_{2y} & u_{2z} \\ u_{3x} & u_{3y} & u_{3z} \\ u_{4x} & u_{4y} & u_{4z} \end{matrix} \right],\#\left( 13 \right) \end{aligned}$$

$$\begin{aligned} \mathbf{J}=\frac{\partial\mathbf{N}}{\partial\boldsymbol{\xi}} \mathbf{u}^{'},\#\left( 14 \right) \end{aligned}$$

$$\begin{aligned} \frac{\partial\mathbf{N}}{\partial\mathbf{x}}=\mathbf{J}^{-1}\frac{\partial\mathbf{N}}{\partial\boldsymbol{\xi}}\boldsymbol{.\#}\left( 15 \right) \end{aligned}$$

In Eq. (15), the transformation between the real space $\mathbf{x}$ and the reference space $\boldsymbol{\xi}$ allows computation of the strain-displacement matrix **B**, which is used in the element stiffness matrix $\mathbf{D}$ assembly as in Eq. (16):

$$\begin{aligned} \mathbf{D}=\frac{\det\left( \mathbf{J} \right)}{6}\int B^{T}\mathbf{C}B dV\boldsymbol{.\#}\left( 16 \right) \end{aligned}$$

The contributions of $\mathbf{D}$ are assembled into the global stiffness matrix $\mathbf{K}$ via standard finite element procedures using the element’s connectivity array. Upon applying the load force $\mathbf{F}$, the displacement field $\mathbf{u}$ can be directly computed using Eq. (17).

$$\begin{aligned} \mathbf{F=K}\mathbf{u}\boldsymbol{\#}\left( 17 \right) \end{aligned}$$

**Numerical simulations**

In numerical simulations, our implementation includes two steps. First, we update the displacement $\mathbf{u}$ by using the Lagrangian mentioned above. Second, the coupled evolution of all field variables, such as the director $\mathbf{n}$, the displacement field $\mathbf{u}$, and the order parameter $s$, is then computed by the gradient descent dynamics based on the Euler–Lagrange equation. Specifically, each variable evolves toward the local minimum of free energy density $f_{\mathrm{tot}}$ through a relaxation process based on the temporal evolution equations:

$$\begin{aligned} \frac{\partial n_{i}}{\partial t}=-\frac{1}{\eta}\left\{ \frac{\partial f}{\partial n_{i}}-\sum_{j} \frac{\partial}{\partial j}\left[ \frac{\partial f}{\partial\left( \partial_{j}n_{i} \right)} \right] \right\},\#\left( 18 \right) \end{aligned}$$

$$\begin{aligned} \frac{\partial u_{i}}{\partial t}=-\frac{1}{\eta}\left\{ \frac{\partial f}{\partial u_{i}}-\sum_{j} \frac{\partial}{\partial j}\left[ \frac{\partial f}{\partial\left( \partial_{j}u_{i} \right)} \right] \right\},\#\left( 19 \right) \end{aligned}$$

$$\begin{aligned} \frac{\partial s}{\partial t}=-\frac{1}{\eta}\left\{ \frac{\partial f}{\partial s}-\sum_{j} \frac{\partial}{\partial j}\left[ \frac{\partial f}{\partial\left( \partial_{j}s \right)} \right] \right\}.\#\left( 20 \right) \end{aligned}$$

Here, $\eta$ is the effective viscosity coefficient of the dynamics that controls the rate of relaxation. The computation is repeated through the first and second steps, where these equations are integrated iteratively over time until convergence is reached.

To numerically simulate the embossing induced orientational, we employ the cuboid model (1.2 mm × 1.2 mm × 0.3 mm) with a mesh of 37 × 37 × 31 to simulate the change of the deformation $u_{i}$, the orientation $n_{i}$ and the order parameter $s$. At first, the cuboid is placed on a rigid substrate, which constrains only the displacement in the z-direction at the bottom surface. All other translational degrees of freedom remain free. The cuboidal elastomer is in the isotropic phase at the initial state. Consequently, the order parameter $s$ is uniformly set to zero across the entire domain, and the director field is also initialized with random orientation. Then, a gradually increasing vertical compressive force is applied through a cylindrical stamp with a diameter of 0.25 mm at the center of the top surface. After each incremental loading step, the displacement field $u_{i}$ is first updated using the Lagrangian by solving the linear system assembled from the global stiffness matrix. Subsequently, the Euler–Lagrange method is employed to relax all field variables, including the deformation field $u_{i}$, the director field $n_{i}$and the order parameter $s$, toward the local free energy minimum. This two-step process—mechanical deformation followed by variational relaxation—is iteratively repeated for each successive loading increment. This iterative scheme ensures that the system gradually evolves toward a mechanically and thermodynamically consistent equilibrium configuration under increasing compressive loading.

After the LCE deformation and orientational equilibrate at specific the compression condition, the director field is fixed by photo- polymerization process as in the experiment. This locking of the anisotropic structure shifts the equilibrium state of the order parameter, effectively biasing the system toward the mechanically deformed configuration. As a result, the stress-induced alignment present at the moment of photo-polymerization is retained in the material. To incorporate this effect into the free energy formulation, we introduce the previously generated stress distribution as a constant term $\sigma_{v_{0}}$ in the modified Landau free energy expression:

$$\begin{aligned} f_{\mathrm{ModLand}}=\frac{A}{2} \mathrm{Tr}\left( \mathbf{Q}^{2} \right)+\frac{B}{4} \left[ \mathrm{Tr}\left( \mathbf{Q}^{2} \right) \right]^{2}-\frac{C}{\mu}\left( \sigma_{v}+\sigma_{v_{0}} \right)\mathrm{Tr}\left( \mathbf{Q} \right)\#\left( 21 \right) \end{aligned}$$

Here, $\sigma_{v_{0}}$ represents the "frozen-in" residual stress arising from the mechanically deformed state at the time of photo-polymerization. In the subsequent heating simulation, the director field and order parameter distribution resulting from the compressive deformation are adopted as the initial state. In the heating simulation, the temperature is increased step by step. After each temperature increment, all field variables—including the displacement field, director field, and order parameter—are relaxed using the Euler–Lagrange method until a steady state is reached. This process is repeated iteratively with further temperature increases, until the scalar order parameter decreases to a sufficiently low value, indicating the approach to an isotropic state.

**The eigensystem of strain tensor** $\boldsymbol{\varepsilon}$ **and its physical meaning:**

To better understand the coupling between the deformation gradient $\boldsymbol{\lambda}_{t}$ and the director $\mathbf{n}$ in the mechanical elastic energy $f_{\mathrm{ME}}$, it is significant to clarify the concept of the strain tensor $\boldsymbol{\varepsilon}$—similar to the second term in the deformation gradient—is often used to describe complex strain states in engineering mechanics. The strain tensor can be written as:

$$\begin{aligned} \boldsymbol{\varepsilon=}\frac{1}{2}\left[ \nabla\mathbf{u+}\left( \nabla\mathbf{u} \right)^{T} \right]\mathbf{=}\left[ \begin{matrix} \varepsilon_{xx} & \varepsilon_{yx} & \varepsilon_{zx} \\ \varepsilon_{xy} & \varepsilon_{yy} & \varepsilon_{zy} \\ \varepsilon_{xz} & \varepsilon_{yz} & \varepsilon_{zz} \end{matrix} \right]\boldsymbol{\#}\left( 22 \right) \end{aligned}$$

Solving the eigensystem of strain tensor $\boldsymbol{\varepsilon}$is a common method for identifying the direction of maximum elongation and compression in each element after the system undergoes complicated deformation. It is evident that the strain tensor has three eigenvectors $\mathbf{v}_{1}$, $\mathbf{v}_{2}$, and $\mathbf{v}_{3}$, with corresponding eigenvalues $\lambda_{1}$, $\lambda_{2}$, and $\lambda_{3}$. The three mutually perpendicular eigenvectors $\mathbf{v}$ define the principal deformation direction of an object under applied load, with the corresponding eigenvalues $\lambda$ indicating the type of deformation way, tension for $\lambda>0$, unchanged for $\lambda=0$ and compression for $\lambda<0$ along the respective directions.

In the Neo-classical theory, director fields prefer to align parallel to the direction of maximum elongation, which corresponds to the eigenvector $\mathbf{v}_{\max}$ associated with the largest eigenvalue $\lambda_{\max}$. Thus, investigating the distribution of the maximum elongation direction $\mathbf{v}_{\max}$ serves as an effective approach to understanding the formation of the director field; the value of $\lambda_{\max}$ is often employed as a means to quantify the coupling between deformation and director field formation.

**Analytical Solution: Stretching/Compression-Induced Reorientation**

To analyze the LCE deformation analytically, only the mechanical elastic free energy $f_{\mathrm{ME}}$ defined in Eq. (1) is considered, which is the primary contribution governing the deformation of the LCE, while other energy terms are neglected.

The experimental observations on stamping-induced deformations have found that the initial state of the LCE is isotropic. Thus, we simplify the initial step length tensor $\mathcal{l}_{0}$ to an isotropic form $\mathcal{l}_{0}=\mathcal{l}_{\perp0} \boldsymbol{\delta}$. Then, we obtain $f_{\mathrm{ME}}$ as below:

$$\begin{aligned} f_{\mathrm{ME}}=\frac{1}{2}\mu\mathrm{Tr}\left( \mathcal{l}_{0}\cdot{\boldsymbol{\lambda}_{t}}^{T}\cdot\mathcal{l}^{-1}\cdot\boldsymbol{\lambda}_{t} \right) \\ =\frac{\mu\mathcal{l}_{\perp0}}{2} \mathrm{Tr}\left[ \boldsymbol{\delta}\cdot{\boldsymbol{\lambda}_{t}}^{T}\cdot\mathcal{l}^{-1}\cdot\boldsymbol{\lambda}_{t} \right]\#\#\left( 23 \right) \end{aligned}$$

To simplify the equation, we perform an approximation of $\mathcal{l}^{-1}$ in terms of the Neumann series expansion of the matrix inverse:

$$\begin{aligned} \mathcal{l}^{-1}&=\mathcal{l}_{\perp}^{-1}\left( \boldsymbol{\delta}+\alpha s \mathbf{nn} \right)^{-1} \\ &=\frac{1}{\mathcal{l}_{\perp}}\left[ \boldsymbol{\delta}-\alpha s\mathbf{nn+}\alpha^{2}s^{2}\left( \mathbf{nn} \right)\left( \mathbf{nn} \right)\boldsymbol{-\ldots} \right] \\ &=\frac{1}{\mathcal{l}_{\perp}}\boldsymbol{\delta}+\frac{-\alpha s+\alpha^{2}s^{2}-\ldots}{\mathcal{l}_{\perp}}\mathbf{nn}\#\left( 24 \right) \end{aligned}$$

Upon inserting Eq. (24) into Eq. (23), we obtain:

$$\begin{aligned} f_{\mathrm{ME}}&=\frac{\mu\mathcal{l}_{\perp0}}{2} \mathrm{Tr}\left\{ \boldsymbol{\delta}\cdot{\boldsymbol{\lambda}_{t}}^{T}\cdot\left[ \frac{1}{\mathcal{l}_{\perp}}\boldsymbol{\delta}+\frac{-\alpha s+\alpha^{2}s^{2}-\ldots}{\mathcal{l}_{\perp}}\mathbf{nn} \right]\cdot\boldsymbol{\lambda}_{t} \right\} \\ &=\frac{\mu\mathcal{l}_{\perp0}}{2} \mathrm{Tr}\left\{ \boldsymbol{\delta}\cdot{\boldsymbol{\lambda}_{t}}^{T}\cdot\left[ \frac{1}{\mathcal{l}_{\perp}}\boldsymbol{\delta} \right]\cdot\boldsymbol{\lambda}_{t}+\underset{=}{\boldsymbol{\delta}}\cdot{\boldsymbol{\lambda}_{t}}^{T}\cdot\left( \frac{-\alpha s+\alpha^{2}s^{2}-\ldots}{\mathcal{l}_{\perp}} \right)\mathbf{nn}\cdot\boldsymbol{\lambda}_{t} \right\} \\ &=\frac{\mu\mathcal{l}_{\perp0}}{2} \mathrm{Tr}\left\{ \frac{1}{\mathcal{l}_{\perp}} {\boldsymbol{\lambda}_{t}}^{T}\cdot\boldsymbol{\lambda}_{t}+\left( \frac{-\alpha s+\alpha^{2}s^{2}-\ldots}{\mathcal{l}_{\perp}} \right){\boldsymbol{\lambda}_{t}}^{T}\cdot\mathbf{nn}\cdot\boldsymbol{\lambda}_{t} \right\} \\ &=\frac{\mu\mathcal{l}_{\perp0}}{2\mathcal{l}_{\perp}} \text{Tr}\left( {\boldsymbol{\lambda}_{t}}^{T}\cdot\boldsymbol{\lambda}_{t} \right)+\frac{\mu\mathcal{l}_{\perp0}}{2\mathcal{l}_{\perp}}\left( -\alpha s+\alpha^{2}s^{2}-\ldots\right)\mathrm{Tr}\left[ {\boldsymbol{\lambda}_{t}}^{T}\cdot\mathbf{n}\boldsymbol{\bigotimes}\mathbf{n}\cdot\boldsymbol{\lambda}_{t} \right] \\ &=\frac{\mu\mathcal{l}_{\perp0}}{2\mathcal{l}_{\perp}} \text{Tr}\left( \left| \boldsymbol{\lambda}_{t} \right|^{2} \right)+\frac{\mu\mathcal{l}_{\perp0}}{2\mathcal{l}_{\perp}}\left( -\alpha s+\alpha^{2}s^{2}-\ldots\right)\left| \boldsymbol{\lambda}_{t}\cdot\mathbf{n} \right|^{2}\#\left( 25 \right) \end{aligned}$$

If the system is isotropic, only the first term remains. We obtain the deformation gradient and the free energy as:

$$\begin{aligned} \boldsymbol{\lambda}_{t}&=\mathrm{diag}\left( \lambda,\frac{1}{\sqrt{\lambda}},\frac{1}{\sqrt{\lambda}} \right)\#\left( 26 \right) \\ f_{\mathrm{IsoME}}&=\frac{\mu\mathcal{l}_{\perp0}}{2\mathcal{l}_{\perp}} \text{Tr}\left( \left| \boldsymbol{\lambda}_{t} \right|^{2} \right) \\ &=\frac{\mu\mathcal{l}_{\perp0}}{2\mathcal{l}_{\perp}}\left( \lambda^{2}+\frac{2}{\lambda} \right)\#\left( 27 \right) \end{aligned}$$

Under the assumption of uniaxiality (along *x*-direction) and incompressible deformation (i.e., $\det\boldsymbol{\lambda}_{t}=1$), the mechanical free energy density in the isotropic state, $f_{\mathrm{IsoME}}$, shows its minimum when $\lambda=1$, corresponding to the situation where the elastomer remains undeformed without external forces. Any deviation from this state, whether extension or compression, leads to an increase in $f_{\mathrm{IsoME}}$.

Now, we move to a more complicated case for anisotropic states where the coupling between the director field $\mathbf{n}$ and the deformation gradient $\boldsymbol{\lambda}_{t}$ must be considered. Assuming the appearance of an infinitesimal nematic order arising from an isotropic background, i.e., $\alpha s\ll1$, the higher-order terms in Eq. (25) can be neglected, leading to the following simplified expression for anisotropic nematic states:

$$\begin{aligned} f_{\mathrm{AniME}}&=\frac{\mu\mathcal{l}_{\perp0}}{2\mathcal{l}_{\perp}} \text{Tr}\left( \left| \boldsymbol{\lambda}_{t} \right|^{2} \right)-\frac{\alpha s\mu\mathcal{l}_{\perp0}}{2\mathcal{l}_{\perp}}\left| \boldsymbol{\lambda}_{t}\cdot\mathbf{n} \right|^{2} \\ &=\frac{\mu\mathcal{l}_{\perp0}}{2\mathcal{l}_{\perp}}\left( \lambda^{2}+\frac{2}{\lambda} \right)-\frac{\alpha s\mu\mathcal{l}_{\perp0}}{2\mathcal{l}_{\perp}}\left[ \lambda^{2}n_{x}^{2}+\frac{1}{\lambda}\left( n_{y}^{2}+n_{z}^{2} \right) \right] \\ &=\frac{\mu\mathcal{l}_{\perp0}}{2\mathcal{l}_{\perp}}\left( \lambda^{2}+\frac{2}{\lambda} \right)-\frac{\alpha s\mu\mathcal{l}_{\perp0}}{2\mathcal{l}_{\perp}}\left[ \lambda^{2}n_{x}^{2}+\frac{1}{\lambda}\left( 1-n_{x}^{2} \right) \right]\#\left( 28 \right) \end{aligned}$$

For a prolate polymer system ($\alpha>0$), the mechanical free energy density $f_{\mathrm{AniME}}$ is minimized when the director aligns along the stretching direction parallel to the x-axis. On the other hand, under compressive deformation along the *x*-axis, $f_{\mathrm{AniME}}$ is minimized when the director tends to lie within the *yz*-plane, perpendicular to the direction of compressing. In contrast, for an oblate polymer system ($\alpha<0$), the behavior is reversed.

**Analytical Solution: Temperature-Induced Reorientation**

Experimentally, LCE stabilizes its deformed configuration after the photo-polymerization process. When heated, LCE transforms to a flattened state with an isotropic orientational configuration. In theory, we can again go to Eq. (1) to analyze the orientation and the deformation. As LCE is isotropic at high temperatures, the current step length tensor $\mathcal{l}$ reduces to an isotropic form $\mathcal{l}=\mathcal{l}_{\perp} \boldsymbol{\delta}$. Thus, the free energy expression $f_{\mathrm{ThME}}$ can be reduced as:

$$\begin{aligned} f_{\mathrm{ThME}}&=\frac{1}{2}\mu\mathrm{Tr}\left( \mathcal{l}_{0}\cdot{\boldsymbol{\lambda}_{t}}^{T}\cdot\mathcal{l}^{-1}\cdot\boldsymbol{\lambda}_{t} \right) \\ &=\frac{1}{2}\mu\mathrm{Tr}\left[ \mathcal{l}_{0}\cdot{\boldsymbol{\lambda}_{t}}^{T}\cdot\left( \mathcal{l}_{\perp} \boldsymbol{\delta} \right)^{-1}\cdot\boldsymbol{\lambda}_{t} \right] \\ &=\frac{\mu}{2\mathcal{l}_{\perp}} \mathrm{Tr}\left[ \mathcal{l}_{\perp0} \left( \boldsymbol{\delta}+\alpha s_{0} \mathbf{n}_{\mathbf{0}}\mathbf{n}_{\mathbf{0}} \right)\cdot{\boldsymbol{\lambda}_{t}}^{T}\cdot\boldsymbol{\delta}\cdot\boldsymbol{\lambda}_{t} \right] \\ &=\frac{\mu\mathcal{l}_{\perp0}}{2\mathcal{l}_{\perp}}\mathrm{Tr}\left[ \boldsymbol{\delta}\cdot{\boldsymbol{\lambda}_{t}}^{T}\cdot\boldsymbol{\delta}\cdot\boldsymbol{\lambda}_{t} \right]+\frac{\mu\mathcal{l}_{\perp0}}{2\mathcal{l}_{\perp}}\mathrm{Tr}\left[ \alpha s_{0} \mathbf{n}_{\mathbf{0}}\mathbf{n}_{\mathbf{0}}\cdot{\boldsymbol{\lambda}_{t}}^{T}\cdot\boldsymbol{\delta}\cdot\boldsymbol{\lambda}_{t} \right] \\ &=\frac{\mu\mathcal{l}_{\perp0}}{2\mathcal{l}_{\perp}}\mathrm{Tr}\left[ {\boldsymbol{\lambda}_{t}}^{T}\cdot\boldsymbol{\lambda}_{t} \right]+\frac{\alpha s_{0}\mu\mathcal{l}_{\perp0}}{2\mathcal{l}_{\perp}}\mathrm{Tr}\left[ \mathbf{n}_{\mathbf{0}}\mathbf{n}_{\mathbf{0}}\cdot{\boldsymbol{\lambda}_{t}}^{T}\cdot\boldsymbol{\lambda}_{t} \right] \\ &=\frac{\mu\mathcal{l}_{\perp0}}{2\mathcal{l}_{\perp}} \text{Tr}\left( \left| \boldsymbol{\lambda}_{t} \right|^{2} \right)+\frac{\alpha s_{0}\mu\mathcal{l}_{\perp0}}{2\mathcal{l}_{\perp}}\mathrm{Tr}\left[ \boldsymbol{\lambda}_{t}\cdot\mathbf{n}_{\mathbf{0}}\mathbf{n}_{\mathbf{0}}\cdot\boldsymbol{\lambda}_{t} \right] \\ &=\frac{\mu\mathcal{l}_{\perp0}}{2\mathcal{l}_{\perp}} \text{Tr}\left( \left| \boldsymbol{\lambda}_{t} \right|^{2} \right)+\frac{\alpha s_{0}\mu\mathcal{l}_{\perp0}}{2\mathcal{l}_{\perp}}\left| \boldsymbol{\lambda}_{t}\cdot\mathbf{n}_{\mathbf{0}} \right|^{2}\#\left( 29 \right) \end{aligned}$$

The Eq. (29) is similar to Eq. (28). The only difference is the sign in the second term. This means that, upon heating after the polymerization, any non-zero nematic order parameter will increase the system’s energy, thus being not favored. Therefore, the system should go back to isotropic state, accompanied by a flattened LCE configuration.

**Figure S10.** (((a) A cuboidal elastomer (1.2 mm × 1.2 mm × 0.3 mm) indented by a cylindrical probe. Compression generates a central surface depression and induces orientational order through stress-order parameter (OP) coupling in landau free energy term. Upon heating, the OP vanishes, the alignment becomes disordered, and the structure nearly recovers its initial shape. (b) Director fields as black arrows and strain distribution ε as the background color, showing ε_xx_ (top), ε_yy_ (middle), and ε_zz_ (bottom) in the xy cross-sections at z = 0, 0.2, and 0.3 mm. All cross-sections are referenced to the initial elastomer geometry (undeformed grid). Coordinates (x, y, z) are expressed in millimeters.))

**Measurement of Birefringence and Director Field**

The birefringence and director field of the LCE samples were measured using a commercial birefringence imaging system (LCC7201B, Thorlabs Inc.). This system is designed to quantify the in-plane optical retardance and fast-axis azimuth angle of birefringent samples with high spatial resolution. All measurements were performed at an operational wavelength $\lambda$= 633 nm with a spatial resolution of 9.77 mm. Samples were placed on the sample stage using an appropriate holder to ensure flat alignment within the imaging area. For each sample, the optical retardance $\delta$ was recorded. From the defining equation of optical retardance, the birefringence $\Delta n$ can be calculated as

$$\begin{aligned} \Delta n=\frac{\delta\cdot\lambda}{2\pi d}.\#\left( 30 \right) \end{aligned}$$

$d$ is the thickness of the LCE sample.

To determine the director field of the LCE sample, we employed a right-handed circularly polarized light beam as the incident sources. The transmitted light from the sample was passed through linear polarizers oriented at 0°, 45°, 90°, and 135°, and the corresponding transmitted intensities were recorded. Based on the Jones matrix formalism [1], the intensity of light passing through each polarizer direction is given by:

$$\begin{aligned} I_{0}=\frac{I_{b}}{2}\left( 1+\sin2\theta\cdot\sin\delta\right)\#\left( 31 \right) \end{aligned}$$

$$\begin{aligned} I_{45}=\frac{I_{b}}{2}\left( 1-\cos2\theta\cdot\sin\delta\right)\#\left( 32 \right) \end{aligned}$$

$$\begin{aligned} I_{90}=\frac{I_{b}}{2}\left( 1-\sin2\theta\cdot\sin\delta\right)\#\left( 33 \right) \end{aligned}$$

$$\begin{aligned} I_{135}=\frac{I_{b}}{2}\left( 1+\cos2\theta\cdot\sin\delta\right)\#\left( 34 \right) \end{aligned}$$

$I_{b}$ denotes the intensity of the incident light. $\theta$ represents the azimuthal angle of the director, and $\delta$ is the optical retardance. Using these intensity measurements, the azimuthal angle $\theta$ can be calculated as:

$$\begin{aligned} \theta=\frac{1}{2}\tan^{-1} \left( \frac{I_{0}-I_{90}}{I_{135}-I_{45}} \right)\#\left( 35 \right) \end{aligned}$$

References

[1] M. Warner and E. M. Terentjev, Liquid Crystal Elastomers. *Oxford University Press* (2003)

**Device integration**

**Figure S11.** (((a)Schematic image of the printed circuit board that was used to demonstrate the localized actuation of the tactile material.))

The demonstration device consists of an 8x8 matrix of chip resistors paired with diodes. By selectively connecting the rows and columns of this grid to a power source, specific resistors can be heated. The LCE with bilayer is attached to this PCB using thermal paste for adhesion and thermal dissipation, placing each programmed dent on a resistor.

**Heating over extended time**

**Figure S12.** ((Thermal footage of the top of the tactile surface before and after locally heating a resistor.))

**Finite Element Method (FEM) analysis of haptic surface**

COMSOL Multiphysics (version 6.1) was used to simulate the electro-thermal behavior of the LCE-based haptic actuator. A three-dimensional multi-physics model was developed by coupling two physics modules: the Electric Currents interface and the Heat Transfer in Solids interface, linked via the Joule Heating Multiphysics coupling. This approach enabled the calculation of ohmic heat generation within the resistive element and the resulting temperature distribution across the multilayer structure.

Two configurations were considered. (1) Without cooling sink: The model geometry consisted of five stacked domains (from bottom to top): a flexible PCB layer (polyimide, 20 × 20 × 0.1 mm³), an SMD resistor (0.2 × 0.4 × 0.13 mm³), a support layer (20 × 20 × 0.13 mm³) for flattening the surface, a LCE layer (20 × 20 × 0.5 mm³), and a protective TPGDA/DODT top layer (20 × 20 × 0.1 mm³). (2) With cooling sink: an aluminum block (20 × 20 × 4 mm³) was added beneath the PCB to serve as a heat sink (Figure S13).

**Figure S13.** ((Geometry used for finite element modelling, without heat sink (left) and with heat sink (right).))

For the Electric Currents interface, the SMD resistor was assigned an electrical conductivity of 1538 S/m, corresponding to a total resistance of 10 Ω. A terminal boundary condition with an applied voltage (1–3 V) and a ground boundary (0 V) were set at the two ends of the resistor, respectively. For the Heat Transfer in Solids interface, the LCE layer was assigned a density of 1200 kg/m³, a specific heat capacity of 820 J(kg·K)^-1^, and a thermal conductivity of 0.2 W(m·K)-1. The TPGDA/DODT layer was assigned a density of 1200 kgm-3, a specific heat capacity of 1700 J(kg·K)^-1^, and a thermal conductivity of 0.1 W(m·K)^-1^. To model heat dissipation to the ambient air, convective heat flux boundary conditions were applied to all external surfaces, with a heat transfer coefficient of 15 W/(m²·K) for natural convection. The ambient temperature was set to 25 °C.

The model was solved using a Time-Dependent solver for transient thermal analysis, with a time step of 0.5 s over a total period of 20 s. The linear system at each iteration was solved using the Conjugate Gradients iterative solver with left preconditioning. The residual tolerance was set to 0.001, and the maximum number of iterations was 10000. A nonlinear-based error norm was enabled to ensure convergence of the coupled electro-thermal problem. Post-processing was performed to extract: (i) the volume-averaged temperature of the 2 × 2 × 0.5 mm³ LCE region (**Figure S14** a); (ii) a 10 mm cut-line on the top surface of the LCE layer to analyze the lateral temperature distribution (Figure S14 b,c); and (iii) a 2D cut-plane through the central axis to visualize the cross-sectional temperature profile (**Figure S14 d,e, Video S1, S2**).

**Figure S14.** ((Results of the Finite Element Modelling. (a) Average temperature over time in 2 x 2 x 0.5 mm volume of LCE directly above the resistor. Temperature profiles of the top surface of the LCE material, underneath the acrylate layer, directly above the resistor (b) in the presence of a heatsink, (c) in the absence of a heatsink. Temperature gradient in 2D in a cross section of the simulated system after 20 s of heating (d) with heatsink, (e) without heatsink.))

**Energy requirements and efficiency**

To quantitatively assess the energy requirement for local actuation, we define the ‘effective energy’ as the net thermal energy absorbed by the targeted LCE volume (2 × 2 × 0.5 mm³) to raise its temperature from 25 °C to 45 °C. Based on the material properties used in our calibrated model (ρ = 1200 kg/m³, Cp = 820 J/(kg·K)), this thermodynamic minimum is calculated as:

$E_{\mathrm{net}}=\rho\cdot V\cdot C_{p}\cdot\Delta T=1200\times\left( 2\times{10}^{-9} \right)\times820\times20\approx0.039\text{ J}$.

However, the total electrical energy drawn from the power source is obtained by integrating the Joule heat over the required heating period: $E_{\mathrm{input}}=\int ec.Qh\text{ }\mathrm{dt}$.

In the case without cooling, over the 8-second heating period (under 1 V and 0.1 A, corresponding to a constant resistance of 10 Ω) this results in a required energy input of: $E_{input}\approx0.8\text{ J}$. This yields an energy efficiency of ~5%, indicating that ~95% of the input energy is dissipated through convection and conduction to the surroundings and underlying layers. The analysis identifies lateral heat spreading as the dominant loss mechanism, which directly justifies the use of a bottom heat sink to suppress thermal crosstalk and maintain independent actuation.

In the case with cooling, because of the increased heat dissipation to the heat sink, a higher power input of 3V and 0.3A is required, which increases the energy input to: $E_{input}\approx7.2\text{ J}$. This decreases the energy efficiency roughly tenfold to ~0.5%, indicating that ~99.5% of the energy input is dissipated to the surroundings and mainly the underlying heatsink.

**Gradual deformation on device**

**Figure S15.** ((Protrusion from the tactile material when different DC voltages are applied to one resistor to induce Joule heating. Different voltages translate to different equilibrium temperatures in the device, corresponding to different degrees of actuation in the material. This could be leveraged to create areas with different textures in a full-scale device.))
